# Supplementary material for: MtvS1 and MtvS2 Interact with RNA polymerase to regulate the Francisella type V-A CRISPR-Cas system
Source: Nat Commun. 2026 Sep 1;17:10400. doi: 10.1038/s41467-026-77062-6 (PMC13627669; doi:10.1038/s41467-026-77062-6)
Supplement: Supplementary file 1 [file 41467_2026_77062_MOESM12_ESM.pdf]

nature portfolio

Corresponding author(s): Maj Brodmann, Luciano A. Marraffini

Last updated by author(s): Jul 16, 2026

# Reporting Summary

Nature Portfolio wishes to improve the reproducibility of the work that we publish. This form provides structure for consistency and transparency in reporting. For further information on Nature Portfolio policies, see our Editorial Policies and the Editorial Policy Checklist.

## Statistics

For all statistical analyses, confirm that the following items are present in the figure legend, table legend, main text, or Methods section.

| n/a | Confirmed | |
|---|---|---|
| ☐ | ☒ | The exact sample size ($n$) for each experimental group/condition, given as a discrete number and unit of measurement |
| ☐ | ☒ | A statement on whether measurements were taken from distinct samples or whether the same sample was measured repeatedly |
| ☐ | ☒ | The statistical test(s) used AND whether they are one- or two-sided<br>*Only common tests should be described solely by name; describe more complex techniques in the Methods section.* |
| ☒ | ☐ | A description of all covariates tested |
| ☒ | ☐ | A description of any assumptions or corrections, such as tests of normality and adjustment for multiple comparisons |
| ☐ | ☒ | A full description of the statistical parameters including central tendency (e.g. means) or other basic estimates (e.g. regression coefficient) AND variation (e.g. standard deviation) or associated estimates of uncertainty (e.g. confidence intervals) |
| ☐ | ☒ | For null hypothesis testing, the test statistic (e.g. $F$, $t$, $r$) with confidence intervals, effect sizes, degrees of freedom and $P$ value noted<br>*Give P values as exact values whenever suitable.* |
| ☒ | ☐ | For Bayesian analysis, information on the choice of priors and Markov chain Monte Carlo settings |
| ☒ | ☐ | For hierarchical and complex designs, identification of the appropriate level for tests and full reporting of outcomes |
| ☒ | ☐ | Estimates of effect sizes (e.g. Cohen's $d$, Pearson's $r$), indicating how they were calculated |

*Our web collection on statistics for biologists contains articles on many of the points above.*

## Software and code

Policy information about availability of computer code

| | |
|---|---|
| Data collection | NIS-Elements AR version 5.21.03<br>ONIX2 version 1.0.4<br>Gen5 version 3.15.15<br>Miseq version4.0<br>Miseq 100i version 1.0.109<br>NovaSeq X Plus with with bcl-convert version 4.2.4<br>Diomni™Design and Analysis (RUO) version 3.1.0 |
| Data analysis | GraphPad Prism version 10.5.0<br>Fiji version 1.54p<br>SnapGene v8.2.2<br>EDGE-Pro version 1.3.1<br>DeSeq2 version 1.40.2<br>R version 4.4<br>ProteomeDiscoverer version 1.4.1.14 and 3.2<br>Mascot version 2.8 and 3.1<br>MaxQuant version 2.0.3.0 and 2.6.6.9<br>Spectronault version 19.7<br>Perseus (v 1.6.15.0) |

FUNAGE-Pro version 2 (http://funagepro.molgenrug.nl/)
WebLogo3 (https://weblogo.berkeley.edu/create.cgi)
Foldseek Search server (https://search.foldseek.com/search)
AlphaFold 3 server (https://alphafoldserver.com/)
Pymol version 2.5.7
MEGA12 version 12.0.10
heatmap2 (usegalaxy.org, Galaxy Version 3.3.0)
Prodigal version 2.6.3
HMMER version 3.4
DefenseFinder version 1.3.0

For manuscripts utilizing custom algorithms or software that are central to the research but not yet described in published literature, software must be made available to editors and reviewers. We strongly encourage code deposition in a community repository (e.g. GitHub). See the Nature Portfolio guidelines for submitting code & software for further information.

## Data

Policy information about availability of data

All manuscripts must include a data availability statement. This statement should provide the following information, where applicable:

- Accession codes, unique identifiers, or web links for publicly available datasets
- A description of any restrictions on data availability
- For clinical datasets or third party data, please ensure that the statement adheres to our policy

RNA-seq data is deposited at NCBI with Bioproject ID PRJNA1308637
Proteomics data is deposited on PRIDE with ID: PXD068088
Source data is provided as a Source Data File

## Research involving human participants, their data, or biological material

Policy information about studies with human participants or human data. See also policy information about sex, gender (identity/presentation), and sexual orientation and race, ethnicity and racism.

| | |
|---|---|
| Reporting on sex and gender | N/A |
| Reporting on race, ethnicity, or other socially relevant groupings | N/A |
| Population characteristics | N/A |
| Recruitment | N/A |
| Ethics oversight | N/A |

Note that full information on the approval of the study protocol must also be provided in the manuscript.

# Field-specific reporting

Please select the one below that is the best fit for your research. If you are not sure, read the appropriate sections before making your selection.

[x] Life sciences    [ ] Behavioural & social sciences    [ ] Ecological, evolutionary & environmental sciences

For a reference copy of the document with all sections, see nature.com/documents/nr-reporting-summary-flat.pdf

# Life sciences study design

All studies must disclose on these points even when the disclosure is negative.

| | |
|---|---|
| Sample size | As is routine in microbiology, most experiments were performed in biological triplicates, with exact sample sizes reported in figure legends. |
| Data exclusions | No data was excluded. |
| Replication | Experiments were repeated at least twice and were successfully replicated. Western blot and MtvS2-His6 EMSA were only performed once. |
| Randomization | No randomization was carried out as there are no animals or human experiments. Experimental outcome did not depend on the order in which samples were analyzed. |
| Blinding | Blinding was not relevant as there are no animal or human experiments. Knowledge of order or identity of samples did not change experimental outcome. |

# Reporting for specific materials, systems and methods

We require information from authors about some types of materials, experimental systems and methods used in many studies. Here, indicate whether each material, system or method listed is relevant to your study. If you are not sure if a list item applies to your research, read the appropriate section before selecting a response.

## Materials & experimental systems

| n/a | Involved in the study |
|---|---|
| ☐ | ☒ Antibodies |
| ☒ | ☐ Eukaryotic cell lines |
| ☒ | ☐ Palaeontology and archaeology |
| ☒ | ☐ Animals and other organisms |
| ☒ | ☐ Clinical data |
| ☒ | ☐ Dual use research of concern |
| ☒ | ☐ Plants |

## Methods

| n/a | Involved in the study |
|---|---|
| ☒ | ☐ ChIP-seq |
| ☒ | ☐ Flow cytometry |
| ☒ | ☐ MRI-based neuroimaging |

## Antibodies

Antibodies used

primary mouse THE His Tag anti-His6 antibody (GenScript, A00186), diluted 1:4000
secondary goat anti-mouse IgG (H+L) HRP antibody (Thermo Fisher Scientific, 31430), diluted 1:10000

Validation

primary mouse THE His Tag anti-His6 antibody (GenScript, A00186), https://www.genscript.com/antibody/A00186S-THE_His_Tag_Antibody_mAb_Mouse.html
secondary goat anti-mouse IgG (H+L) HRP antibody (Thermo Fisher Scientific, 31430), https://www.thermofisher.com/antibody/product/Goat-anti-Mouse-IgG-H-L-Secondary-Antibody-Polyclonal/31430

## Plants

Seed stocks

N/A

Novel plant genotypes

N/A

Authentication

N/A
