# Supplementary material for: MtvS1 and MtvS2 Interact with RNA polymerase to regulate the Francisella type V-A CRISPR-Cas system
Source: Nat Commun. 2026 Sep 1;17:10400. doi: 10.1038/s41467-026-77062-6 (PMC13627669; doi:10.1038/s41467-026-77062-6)
Supplement: Supplementary file 2 [file 41467_2026_77062_MOESM13_ESM.pdf]

nature portfolio

Peer Review File

MtvS1 and MtvS2 Interact with RNA Polymerase to Regulate the Francisella Type V-A CRISPR-Cas System

Corresponding Author: Dr Luciano A. Marraffini

This file contains all reviewer reports in order by version, followed by all author rebuttals in order by version.

Version 0:

Reviewer comments:

Reviewer #1

(Remarks to the Author)
Major Points:
In Brodmann, et al, the authors explore expression of two co-occurring CRISPR-Cas systems–type II-B and type V-A–in Francisella novicida during different stages of growth. They demonstrate that cas9 is constitutively expressed while cas12 is upregulated during stationary phase. They report two newly identified factors, MtvS1 and MtvS2, that are required for transcriptional upregulation of cas12 at high cell density. They demonstrate that MtvS1 and MtvS2 bind RNA polymerase from E. coli and are predicted to bind RNA polymerase from E. coli and F. novicida. They propose that MtvS1 and MtvS2 are noncanonical alternative sigma factors and show that deletion of MtvS1 affects many different genes and cell pathways in F. novicida, including genes involved in virulence. They suggest that cas12 is upregulated during high cell density–mostly likely in response to quorum sensing–to provide additional defenses when bacteria are more prone to phage infection.

The research is very well done and the models (and alternatives) they propose are rigorous and carefully considered. The authors use both hypothesis-driven and exploratory methods to discover and investigate MtvS1 and MtvS2, and their conclusions are supported by multiple independent assays that are well done and clearly described. The discovery of factors required for CRISPR-Cas regulation (in an experimentally challenging native host, no less) is notable and provides valuable insight into an understudied phenomenon.

Despite these merits, we hesitate to recommend this for publication in Nature Communications simply because the discoveries made are not obviously impactful for a sufficiently broad audience. Although the data showing MtvS1 is required for cas12a upregulation is convincing, MtvS1 does not affect Cas12a immunity in plasmid assays. Perhaps cas12a upregulation does impact immunity during stationary phase in other contexts (e.g. phage infection), which would be worthwhile to test if possible. It's also not clear if MtvS1 is broadly involved in regulation of cas12a or other CRISPR-Cas systems outside of F. novicida. The authors show that MtvS1 affects many different pathways in F. novicida, some more strongly than cas12a, which is a notable result. However, since this isn't explored more, it's unclear how impactful these findings would be for understanding F. novicida biology. Overall, we believe this study is well done and merits publication but defer to the editors on its suitability for publication in Nature Communications.

Minor Points:
• Many of the graphs lost resolution upon PDF conversion and were difficult to see clearly
• Lines 224 – 226 are confusing and should be described more clearly
• It would be worthwhile to discuss the similarity between MtvS1 and YgfB more (e.g. details on percent identity or structural alignment with RMSD).
• The Extended Data Figure 5 figure legend has some swapped descriptions
• Incorrect spelling of locus on line 330
• Figure 4 – presumably should be "raw" not "row"?
• It's surprising that MtvS1 does not bind the cas12a promoter directly, given how it was identified and its effect on cas12a expression. Including the EMSA results—ideally both with and without MtvS2—would be valuable, as this point is central to the mechanism. It's certainly plausible that additional cofactors are required, and identifying such factors would significantly enhance the impact and mechanistic depth of the work.

Reviewer #2

(Remarks to the Author)

The work by Broadmann et al. represents a substantial effort to elucidate the regulation of the Type V-A CRISPR-Cas system in Francisella. The authors report that this CRISPR system is upregulated during stationary phase and under high-cell-density conditions. They identify and characterize two previously unannotated factors, MtvS1 and MtvS2, as responsible for this differential expression. These regulators interact with RNA polymerase subunits, as demonstrated by pull-down assays. This study constitutes a significant and high-quality contribution to understanding CRISPR regulation, an area still underexplored due to the complexity of studying these systems in their native bacterial contexts. Notably, it functionally characterize previously unknown genes that interact with RNA polymerase and may represent noncanonical alternative sigma factors modulating the expression of multiple Francisella genes, including the Type V-A CRISPR-Cas locus. The findings advance our understanding of transcriptional regulation in this opportunistic pathogen. However, the molecular mechanisms by which MtvS1 and MtvS2 modulate transcription remain uncharacterized. Moreover, while the authors suggest an intriguing link between quorum sensing and CRISPR regulation via MtvS1, the evidence provided remains preliminary. Finally, the study does not directly demonstrate how this regulation impacts CRISPR adaptive immunity against mobile genetic elements, limiting the overall functional conclusions.

Major Comments
1. The main concern is that disruption of mtvS1, despite markedly reducing cas12 transcription, does not impair protospacer interference (Extended Data Fig. 2f). Although cas12 transcript levels decrease, Cas12 protein abundance remains high (Fig. 4c), partially explaining why CRISPR immunity is unaffected. This suggests that MtvS1 and MtvS2 may not be the primary regulators of CRISPR activity, and that additional regulatory factors could be involved. Testing interference efficiency in an mtvS1-S2 double mutant would help determine whether these factors act redundantly or synergistically.
At the same time, the absence of an observable effect on interference may result from the experimental conditions used. The current interference assay evaluate cells during exponential growth, whereas the authors propose that the Type V-A system is upregulated under stationary-phase or high-cell-density conditions to enhance adaptive immunity. Thus, the regulation described here is not directly linked to CRISPR immunity, either at the level of interference or adaptation. To address this, the authors could consider mimicking stationary-phase or high-densitity conditions in their interference assays, for example, by performing experiments in spent overnight medium, under nutrient-limited or minimal-medium conditions, or using alternative delivery systems (e.g., transformation or transduction instead of conjugation). Such approaches would substantially strengthen the biological relevance of the model and clarify whether the observed transcriptional regulation indeed influences CRISPR adaptive immunity.
2. The fluorescence microscopy data in Fig. 3 is central to claim that quorum sensing partecipate to the MtvS1 regulation. This data requires quantification of the fluorescent signal across a large sample size (ideally ~500 cells). As currently presented, the images or videos alone do not convincingly support the claimed reduction, especially given the high background autofluorescence.

Minor Comments
• In general, figures resolutions are not high enough.
• Line 47: Citation missing. Please add: Pastuszka, A., Mazzuoli, M.V., Crestani, C. et al. The virulence regulator CovR boosts CRISPR–Cas9 immunity in Group B Streptococcus. Nat Commun 16, 5678 (2025). https://doi.org/10.1038/s41467-025-60871-6.
• Line 155: Please discuss the lack of complementation for cas2, the final gene in the operon. More generally, complementation appears less effective for genes further downstream from cas12, could this be due to transcriptional polarity or instability of the downstream transcripts?
• Figure 1b/c: Dotted lines are not visible.
• Extended Data Fig. 2h: While mtvS1 transcript and protein levels do not vary between exponential and stationary phases, the mtvS1-mCherry fusion shows increased protein levels in stationary phase. Could this reflect enhanced stability of the tagged variant? Please comment.
• Figure 4 legend: Please specify what the Z-score reflects in the legend.
• Lines 214-222: The promoter analysis is not clearly linked to the conclusions. The presence of canonical motifs (TATAAT at –10, a variable –35 region) in both upregulated and downregulated promoters does not seem to distinguish classes of promoters or inform the regulatory mechanism. Please clarify the rationale and implications.
• Line 229: Correct reference to Fig. 4d instead of Fig. 4e.
• Line 225: Correct reference to Fig. 4c instead of Fig. 5d.
• Lines 234-238: The rationale for highlighting specific genes is unclear. Please explain the selection criteria and how these examples support your conclusions.
• Lines 253-254: A standard western blot cannot exclude post-translational modifications such as phosphorylation. Please rephrase accordingly.
• Line 364: The idea that Francisella RNA polymerase may be required for in vitro transcription is intriguing. Have the authors compared the Francisella and E. coli polymerase subunit compositions or sequence divergence to support this hypothesis?

Reviewer #3

(Remarks to the Author)
The study by Brodmann and colleagues reports the regulation of CRISPR-Cas expression in Francisella tularensis. The strain under study contains two CRISPR-Cas systems, of types II-B and V-A, respectively. The authors show that the type II-B system is constitutively expressed, while the type V-A system is induced during stationary phase and at high cell density. The experimental data clearly support these observations, which are in agreement with previous data indicating the upregulation of defence mechanisms in stationary phage, as the authors also indicate (line 319).

In the study of the mechanisms involved in the regulation of the type V-A system, the authors identified two proteins, MtvS1 and MtvS2, and clearly showed that they play a role in transcriptional regulation of that system. This finding is of particular interest because the characterization of proteins related to MtvS1 remains very limited, and no proteins similar to MtvS2 have been characterized to date. The authors have performed a broad range of well-designed experiments using different complementary techniques. As a result, the authors propose that these proteins act as alternative sigma factors. This is an attractive hypothesis supported by some evidences. However, the data provided are not conclusive. In fact, in vitro transcription assays using the E. coli RNA polymerase holoenzyme failed to reconstitute the transcriptional activity. As the authors indicate in the manuscript, different results might have been obtained using the Francisella RNA polymerase, but those experiments have not been reported.

Specific comments.
1. The introduction of the manuscript would have been improved by covering the regulation of CRISPR-Cas systems in a more detailed way. In line 44, the authors mention the differential regulation of coexisting CRISPR-Cas systems and cite reference 14, which does not address this topic. Moreover, listing 21 references in a single sentence (line 47) is not very useful to put into context the study submitted.

2. The quality of several figures in the manuscript should be improved. Some are difficult to interpret clearly. For example, Figures 1b (adding arrows to identify the proteins would help), 1c, 2a, 2b, etc. The figures appear blurred which I wonder if it could be due to file compression during the submission process.

3. Figure 1 legend: It is mentioned that "Dotted horizontal line: p-value of 0.05. Dotted vertical lines: 2-fold difference." However, those dotted lines are not shown in the figure.

4. Line 114: the authors refer to the "small repeat unit (SRU)" while extended data figure 1B used the term "single repeat unit". It is also unclear whether the SRU could have been represented in Fig. 1g. It would have been informative to indicate where SRU is located in relation to the -35 and -10 sites.

5. Line 117: the authors mention that "the fluorescence pattern did not change in absence of cas12 (Fig. 1f)". It would be useful to clarify that these results should be compared with those in Fig. 1a (lower pannels).

6. Lines 123-124: the phrase would improve by indicating clearly that the sequence AAGATT shown is the one detected in the cas12 promoter, and by including the canonical sequence for comparison.

7. The sentences in lines 223-225 are confusing. Please indicate where the data for Cas12 protein levels are shown. The mention of fig. 5d is not correct, if the comparison is at the proteomic level, it seems that Fig, 4d should mentioned.

8. Line 227h the correct figure reference should be Extended data Fig. 2f, not 2b.

9. Line 229: Fig. 4e is mentioned, but this figure doesn't exist (perhaps it should be Fig. 4d).

10. Extended Fig. 5. Please check the legend, as some figure panel references are incorrect. For example, legend "a" refers to panel "B".

11. Sometimes, the data labeling could be improved. For example, in line 138 it isd indicated that FTN_1238 as enriched (Fig. 2b), yet the figure labels it as Mtvs1, a designation first introduced in line 165. Similarly, in lines 293-295 (Fig. 6a), FTN_1519 is mentioned, while the figure uses the MtvS2 name, first introduced in line 296.

12. Line 666: change "transformants" for "transconjugants".

Reviewer #4

(Remarks to the Author)
I co-reviewed this manuscript with one of the reviewers who provided the listed reports. This is part of the Nature Communications initiative to facilitate training in peer review and to provide appropriate recognition for Early Career Researchers who co-review manuscripts.

Version 1:

Reviewer comments:

Reviewer #1

(Remarks to the Author)
In this resubmitted manuscript, Brodmann, et al include valuable new data that explores the functional impact and broader relevance of two proteins, MtvS1 and MtvS2, on CRISPR-Cas expression. Namely, they show that homologs of MtvS1 are widespread in bacteria and impact expression of type I-E CRISPR-Cas systems, supporting a broader role in CRISPR-Cas regulation. They also provide evidence that mutations in MtvS1/2 decrease Cas12a-mediated targeting of plasmids compared to control (albeit with some inherent confounding factors, which the authors explicitly describe). These additions show a broader relevance, which ameliorates some of my concerns. The authors also provide additional data on MtvS2.

The claims are supported by the evidence and the methodologies appear sound. The main issue, raised by myself and other reviewers, is that the impact on CRISPR-Cas expression is mild and the functional impacts of MtvS1 and MtvS2 remain unclear. The data provided in figure 2l provide some support for impact but rely on a compromised Cas12a system and with confounding factors that cannot be avoided. The authors have clearly done a lot of work that is valuable to the field, but it still remains unclear to me how important these transcriptional regulators are for CRISPR-Cas biology, per se, which reduces the significance of the work. Given that E. coli has many phages, it would be interesting to see if YgfB impacts phage targeting by type I-E CRISPR-Cas, given that Francisella lacks known phages. All the same, the characterization of two widespread transcriptional regulators that have broad impacts on bacterial gene expression is a notable contribution to bacteriology, even if the relative impact on CRISPR-Cas expression is limited.

Reviewer #2

(Remarks to the Author)
The revisions made by the authors are very satisfactory, and I believe they have significantly improved the quality of the manuscript. My main concern in the previous version was the lack of an observable effect on Type V-A interference in the mtvS1-2 mutant. However, despite the limitations of the available tools, the authors developed a very clever strategy to increase the dynamic range of the plasmid-targeting assay. This substantially strengthened the experimental evidence.

The other points I raised, some of which were also noted by the other reviewers, have been adequately addressed. In particular, the demonstration of the conservation and activity of MtvS1 homologues in E. coli broadens the relevance of the findings and increases the overall impact of the study.

I strongly support publication of this work in your journal. The regulation of CRISPR systems remains an important and underexplored area, despite the widespread use and application of CRISPR technologies, in large part because these systems are experimentally challenging to study. The methodology used in this manuscript meets high standards in the field, and the experimental work is comprehensive, rigorous, and sound.

Open Access This Peer Review File is licensed under a Creative Commons Attribution 4.0 International License, which permits use, sharing, adaptation, distribution and reproduction in any medium or format, as long as you give appropriate

credit to the original author(s) and the source, provide a link to the Creative Commons license, and indicate if changes were made.
In cases where reviewers are anonymous, credit should be given to 'Anonymous Referee' and the source.
The images or other third party material in this Peer Review File are included in the article’s Creative Commons license, unless indicated otherwise in a credit line to the material. If material is not included in the article’s Creative Commons license and your intended use is not permitted by statutory regulation or exceeds the permitted use, you will need to obtain permission directly from the copyright holder.
To view a copy of this license, visit https://creativecommons.org/licenses/by/4.0/

Rebuttal Letter

We thank all reviewers for evaluating our manuscript and their insightful suggestions. The new version of the manuscript addresses all the issues raised by the referees and therefore we believe that our study have been significantly strengthened by the proposed revisions.

Major additions to the revised manuscript include:

- Addition of *mtvS2 nonsense* mutant RT-qPCR and RNA-seq data (Figure 2g-j, Figure 3b and d, Extended Data Figure 3b, Supplementary Data File, tab F-H)
- We show a small but significant decrease in the efficiency of Cas12-dependent plasmid targeting in the *mtvS1-2 nonsense* mutant (Figure 2l).
- We included a bioinformatic analysis showing that MtvS1 homologs are widespread and often found in genomes also encoding type I and type IV CRISPR-Cas system (Figure 6a, Supplementary Data File, tab L-O).
- We investigated the role of MtvS1 homolog YgfB in *E. coli* and show that it also plays a role in type I-E CRISPR-Cas system expression in stationary phase. Importantly, MtvS1 is able to rescue the *ygfB* mutant phenotype (Figure 6d-f). In addition, we found that YgfB also affects transcription of many *E. coli* genes (Figure 6g-i, Extended Data Figure 6c, Supplementary Data File, tabP-R). These new findings highlight the importance of MtvS1 homologs for transcription in stationary phase not only in *Francisella* but also in *E. coli*.

Please find point by point answers to each issue below.

Reviewer #1 (Remarks to the Author):

Major Points:

In Brodmann, et al, the authors explore expression of two co-occurring CRISPR-Cas systems–type II-B and type V-A–in Francisella novicida during different stages of growth. They demonstrate that cas9 is constitutively expressed while cas12 is upregulated during stationary phase. They report two newly identified factors, MtvS1 and MtvS2, that are required for transcriptional upregulation of cas12 at high cell density. They demonstrate that MtvS1 and MtvS2 bind RNA polymerase from E. coli and are predicted to bind RNA polymerase from E. coli and F. novicida. They propose that MtvS1 and MtvS2 are noncanonical alternative sigma factors and show that deletion of MtvS1 affects many different genes and cell pathways in F. novicida, including genes involved in virulence. They suggest that cas12 is upregulated during high cell density–mostly likely in response to quorum sensing–to provide additional defenses when bacteria are more prone to phage infection.

The research is very well done and the models (and alternatives) they propose are rigorous and carefully considered. The authors use both hypothesis-driven and exploratory methods to discover and investigate MtvS1 and MtvS2, and their conclusions are supported by multiple independent assays that are well done and clearly described. The discovery of factors required for CRISPR-Cas regulation (in an experimentally challenging native host, no less) is notable and provides valuable insight into an understudied phenomenon.

We thank the reviewer for considering our discovery of MtvS1/2 “rigorous”, “notable” and of value to the field.

Despite these merits, we hesitate to recommend this for publication in Nature Communications simply because the discoveries made are not obviously impactful for a sufficiently broad audience.

We respectfully disagree with the reviewer since we believe that both topics of the paper, namely CRISPR-Cas systems and transcriptional control, are areas of study that relate to the research of numerous labs around the world. In addition, our view is in line with that last reviewer comment, i.e., that the proper audience for *Nature Communications* should be defined by the editors of the journal, not reviewers nor authors.

Although the data showing MtvS1 is required for cas12a upregulation is convincing, MtvS1 does not affect Cas12a immunity in plasmid assays. Perhaps cas12a upregulation does impact immunity during stationary phase in other contexts (e.g. phage infection), which would be worthwhile to test if possible.

We agree with the reviewer. Unfortunately, we are not aware of any phage available for infection of *Francisella* species to date. Anti-plasmid Cas12 activity is very efficient even in the *mtvS1-2 ns* mutant and the number of surviving transconjugants are below our detection limit. Therefore, we weakened the type V-A CRISPR response to obtain a higher number of transconjugants and look for an observable decrease of this number in the mutant strain. In other words, we extended the dynamic range of the plasmid targeting assay. First, we promoted a general increase in conjugation efficiency by

inactivating two restriction-modification systems that limit the establishment of the target plasmid. Second, we introduced into the conjugative plasmid two targets with mutations in their seed sequence. These mutations generate mismatches between the spacer:protospacer sequences that reduce Cas12 DNA cleavage efficiency (PMID 27096362), allowing for an increase in plasmid conjugation. In these conditions, type V-A immunity reduces the conjugation efficiency by an order of magnitude. In the *mtvS1-2 ns* mutant, however, no difference in conjugation efficiency is detected between targeting and non-targeting conditions, a result that demonstrates that these alternative sigma factors are important for immunity. Given the large number of genes that are regulated by MtvS1/2, it is not possible for us to determine if the targeting decrease is only due to lower Cas12 expression in the mutant strain. Indeed, a caveat of this targeting assay is that the conjugation efficiency of the non-target plasmid control is lower in the *mtvS1-2 ns* mutant than in the parental strain, most likely due dysregulation of genes controlled by these factors (we speculate that changes in expression of cell envelope components that we found to be regulated by these alternative sigma factors in our RNA-seq and proteomics results, may be responsible for this general decrease of conjugative transfer in the *mtvS1-2 ns* mutant). These results are shown in the new Fig. 2l:

l

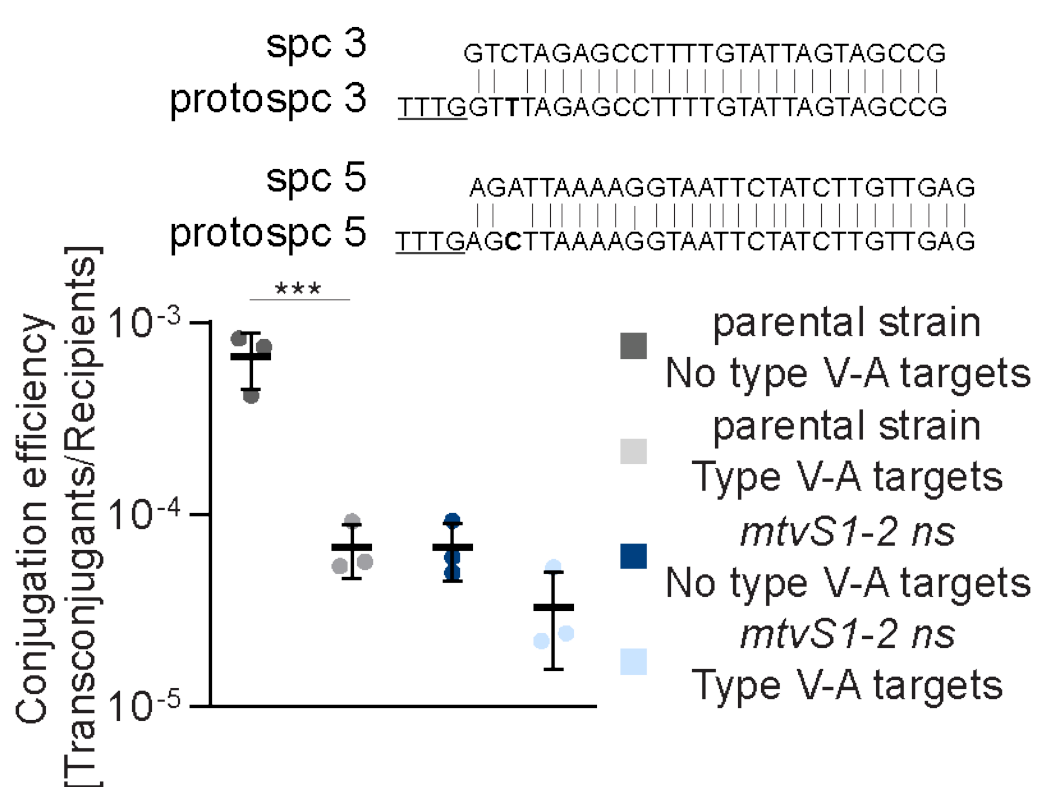

And described in the text as follows:

> "Next, we assessed whether MtvS1-2 dependent regulation of the type V-A CRISPR-Cas locus is important for plasmid restriction. Plasmid transfer into recipients with *mtvS1* and/or *mtvs2* deletions carrying a targeting spacer was not detected, the same as in the case of conjugation into parental recipients (Extended Data Fig. 2m-n). This result prevented us from determining whether the mutations affect plasmid targeting. Therefore, we decided to increase our level of detection of transconjugants to be able to detect differences in anti-plasmid CRISPR immunity, if any. First, we deleted the two restriction enzyme endonucleases, *FTN_0710* and *FTN_1487*, from the parental and mutant strains, which contribute to a low conjugation efficiency. Additionally, we decreased Cas12's targeting activity by introducing mutations in the seed sequence of the protospacer[45]. Since the presence of a single target with a seed sequence mutation completely eliminated immunity, we integrated two protospacers into the conjugative plasmid, matching spacers 3 and 5 of the native type V-A CRISPR array and containing a mismatch at position 3 within each seed sequence (Fig. 2l). In this condition, the conjugation efficiency of a non-target plasmid into the parental strain was increased from $10^{-5}/10^{-4}$ to $\sim 10^{-3}$, and the transfer of a target plasmid produced detectable transconjugants, but with a reduction in conjugation efficiency of one order of magnitude compared to the non-target control (Fig. 2l). In contrast, plasmid targeting did not affect conjugation efficiency into *mtvS1-2 nonsense* mutant recipients (Fig. 2l), a result that indicates that these genes are important for the type V-A CRISPR immune response, presumably due to their effects on *cas12* regulation. Interestingly, conjugation efficiency of the non-target plasmid into *mtvS1-2 nonsense* mutant recipients was

reduced by an order of magnitude when compared to the parental strain, a finding that suggests the involvement of these genes in the regulation of other genes outside of the type V-A CRISPR-*cas* operon (see below) that affect conjugation, possibly involved in the generation of cell wall structures."

It's also not clear if MtvS1 is broadly involved in regulation of cas12a or other CRISPR-Cas systems outside of F. novicida.

We thank the reviewer for prompting us to investigate and compare MtvS1 homologs from other species, as this comment led to very interesting results. First, we found MtvS1 homologs in ~ 40 % of the deposited bacterial genomes and that they are more often encoded in genomes containing Type I and IV CRISPR-Cas systems than in genomes harboring the other CRISPR types (new Fig. 6a):

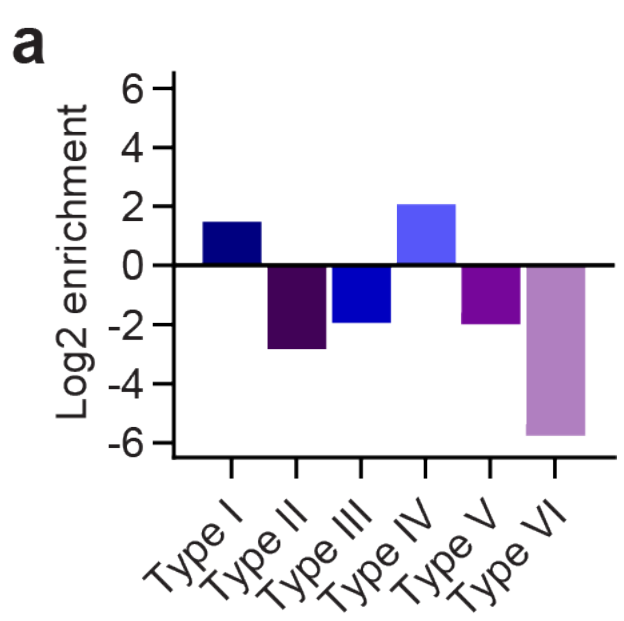

We then tested the role of the *E. coli* MtvS1 homolog, YgfB, and found that disruption of the *ygfB* gene decreased transcription of the type I-E *cas* genes of this organism, as measured by RT-qPCR. Importantly, this decrease was rescued by the expression of either *E. coli* YgfB or *F. novicida* MtvS1, a result that strongly suggests similar functions for these homologs (new Figs. 6d-f):

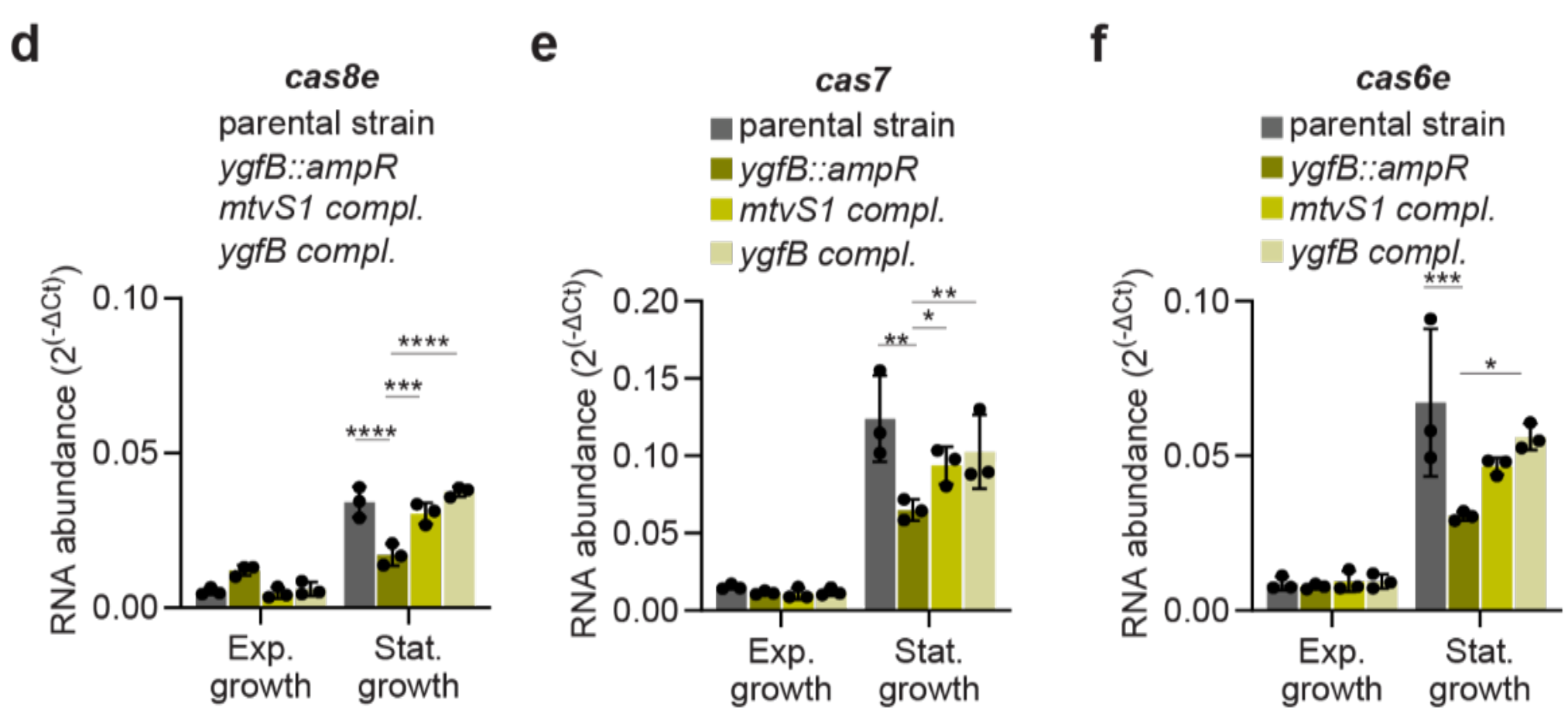

Finally, we performed RNA-seq and detected many genes that were differentially expressed in the *ygfB* mutant compared to the parental strain in stationary phase (new Fig. 6g-i):

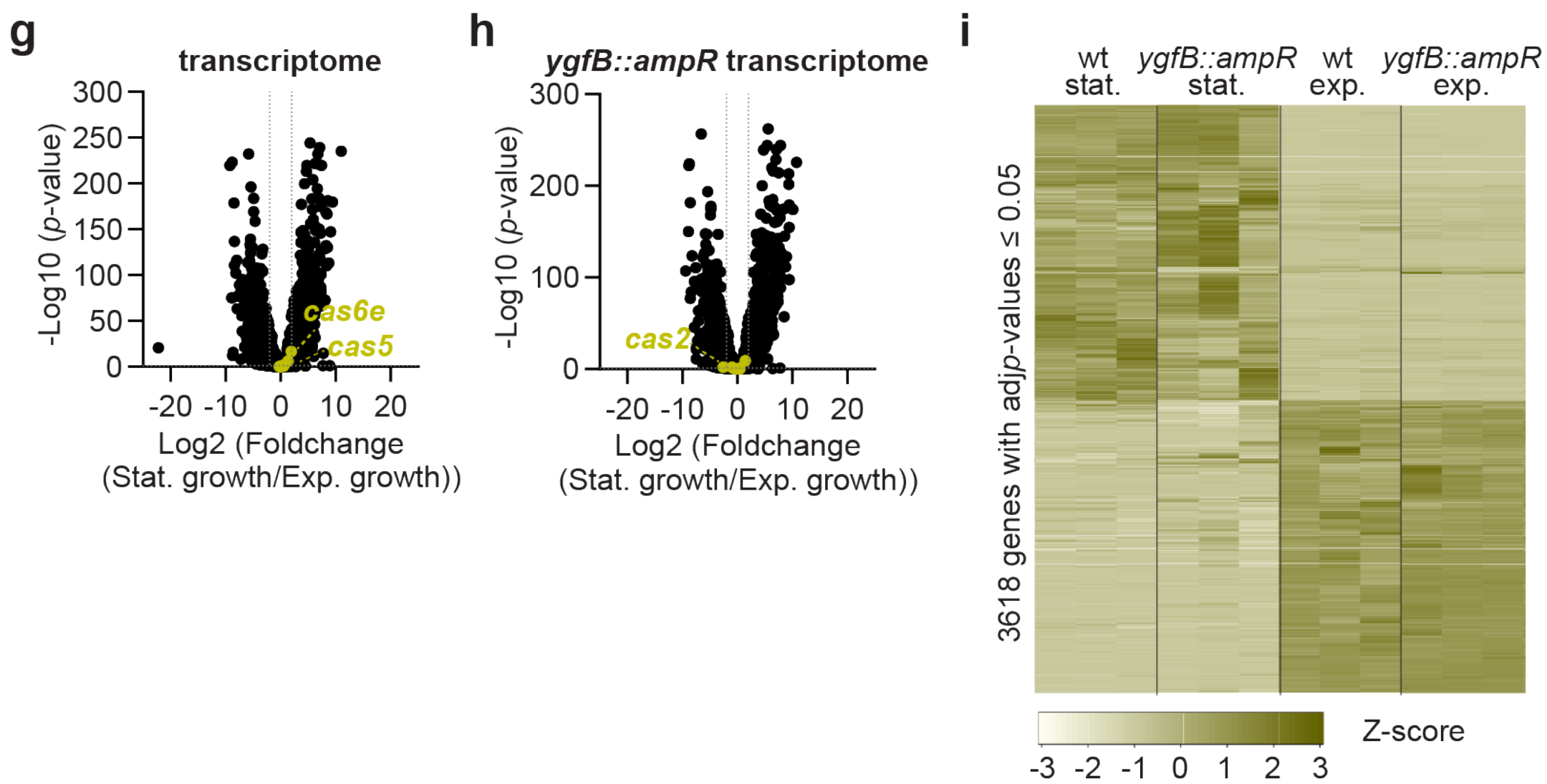

We therefore conclude that MtvS homologs are spread among bacteria, often co-localize with type I and IV CRISPR-Cas systems, and function in the regulation of these loci as well as other genes. The new results are described in a new section of the revised manuscript entitled "*YgfB is a MtvS1 homolog that affects expression of the type I-E CRISPR-Cas system in* E. coli*"*.

YgfB is a MtvS1 homolog that affects expression of the type I-E CRISPR-Cas system in E. coli

We searched for MtvS1 homologs in 99,999 bacterial genomes and found them present in ~ 40 % of the organisms (Supplementary Data File, tab L-O). We also analyzed the distribution of CRISPR-Cas systems within genomes containing MtvS1 homologs and observed that they co-occur more often with Type I and Type IV CRISPR-Cas systems (Fig. 6a, Supplementary Data File, tab L). *E. coli* harbors the MtvS1 homolog YgfB (b2909), which is 27.2 % identical at the amino acid sequence level (Fig. 6b) as well as a Type I-E CRISPR-Cas system[6] (Extended Data Fig. 6b). Structural alignment of the two protein models suggests a highly conserved structure (RSMD value: 1.396 Å over 788 atoms, Fig. 6c, Extended Data Fig. 6a), with many of the MtvS1 residues that are predicted to interact with β' coiled-coil domain conserved in YgfB (Fig. 6b). To investigate whether YgfB affects the transcription of the CRISPR locus, we performed RT-qPCR of the *cas6e*, *cas7* and *cas8e* transcripts, which encode proteins of the Cascade complex involved in crRNA processing and target recognition[77]. We measured RNA levels in both the parental strain and a *ygfB::ampR* mutant that were grown to exponential and stationary phase. Similarly to the results we obtained for the type V-A CRISPR-*cas* locus of *F. novicida*, all transcripts tested were increased in stationary phase cells, compared to exponential growth, for the parental strain (Fig. 6d-f). This increase, however, significantly less pronounced in the *ygfB::ampR* mutant, but was restored in the presence of plasmids expressing YgfB or MtvS1 (using their native promoters) (Fig. 6d-f), a result that suggests that YgfB and MtvS1 have similar functions.

To investigate whether YgfB, like MtvS1, affects the expression of additional genes beyond the *cas* operon, we compared RNA-seq data for the parental strain and *ygfB::ampR* mutant in both growth conditions (Extended Data Fig. 6c, Supplementary Data File, tabs P-R). We found that two genes of the type I-E CRISPR-*cas* operon, *cas6e* and *cas5*, were upregulated in stationary phase (Fig. 6g, Supplementary Data File, tab P). Compared to *cas* transcripts levels in the parental strain in stationary phase, RNA reads for all the genes in the operon, except *cas6e*, were decreased (Fig. 6h, Supplementary Data File, tab P). Beyond the CRISPR-*cas* locus, other genes were differently expressed in the *ygfB::ampR* mutant (Fig. 6i, Supplementary Data File, tab P). GSE analysis revealed stationary phase downregulation of a group of 15 poorly characterized genes (5 of which are encoded in cryptic prophage regions; *b1137*, *b1138*, *b2623*, *b4306*, *b4307*) as well as genes involved in ubiquinone biosynthesis (Supplementary Data File, tab R). On the other hand, expression of carbohydrate transport and metabolism genes was increased in stationary phase in the *ygfB::ampR* mutant. We did not detect significantly up- or down-regulated pathways in

> exponential growth phase compared to the parental strain. Altogether our data showed that MtvS1 and YgfB not only share sequence and structural homology, but also participate in the regulation of *cas* genes and other genes in *F. novicida* and *E. coli*, respectively, most likely through their interaction with RNA polymerase as an alternative sigma factor.

The authors show that MtvS1 affects many different pathways in F. novicida, some more strongly than cas12a, which is a notable result. However, since this isn't explored more, it's unclear how impactful these findings would be for understanding F. novicida biology.

We agree with the reviewer that this is a notable finding worth of further study. However we believe that the investigation of the broader effects of MtvS1/2 regulation are outside of the scope of this manuscript; which centers on the discovery of these alternative sigma factors and their role in Cas12 regulation. We believe that the extended effects of MtvS1/2 activities are important to disclose but investigated in the future. We briefly mention possibilities for the additional regulatory function of MtvS1/2 in the Discussion section as follows:

> "Future studies will investigate the quorum sensing network and the exact mechanisms of how MtvS1 and MtvS2 promote transcription of *cas12* and other genes. Together with the characterization of the MtvS1-2 regulated genes, elucidating these mechanisms in detail will not only be relevant for the understanding phage defense strategies but also for the pathogenesis of *Francisella*."

Overall, we believe this study is well done and merits publication but defer to the editors on its suitability for publication in Nature Communications.

We thank the reviewer for considering our work worthy of publication.

Minor Points:

Many of the graphs lost resolution upon PDF conversion and were difficult to see clearly

We apologize for the inconvenience; we have uploaded original files in this resubmission to avoid this issue.

Lines 224 – 226 are confusing and should be described more clearly

We agree; we edited the text as follows:

> "Last, to corroborate the observed changes in transcription observed in the absence of MtvS1, we performed proteomic analysis of the parental and *mtvS1 nonsense* mutant strains in both growth phases. The data confirmed the increase in Cas12 protein levels when cells enter stationary phase, which was decreased in the absence of MtvS1 (Fig. 1b and 3e, Extended Data Fig. 3d, Supplementary Data File, tab A). Although the decrease of Cas12 levels in the mutant strain is not statistically significant, this result is consistent with the decrease in the Cas12-mCherry2 fluorescence observed using microscopy (Fig. 3f). In spite of this decrease, microscopy showed residual fluorescence signal in the *mtvS1 nonsense* mutant, which was also confirmed by whole-cell proteomics results that revealed a small but detectable increase of Cas12 protein levels in stationary phase compared to exponential growth in this mutant (Fig. 3e-f, Extended Data Fig. 3d, Supplementary Data File, tab A). This result provides a possible explanation for the high Cas12-dependent plasmid targeting levels detected for the *mtvS1* mutant strain (Extended Data Fig. 2m).

Other, statistically significant, changes in protein levels were observed in the absence of MtvS1 (Fig. 3e and g, Extended Data Fig. 3d)."

It would be worthwhile to discuss the similarity between MtvS1 and YgfB more (e.g. details on percent identity or structural alignment with RMSD).

We agree with the reviewer. We have included the structural alignment between MtvS1 and *E. coli* YgfB in the new Fig. 6c:

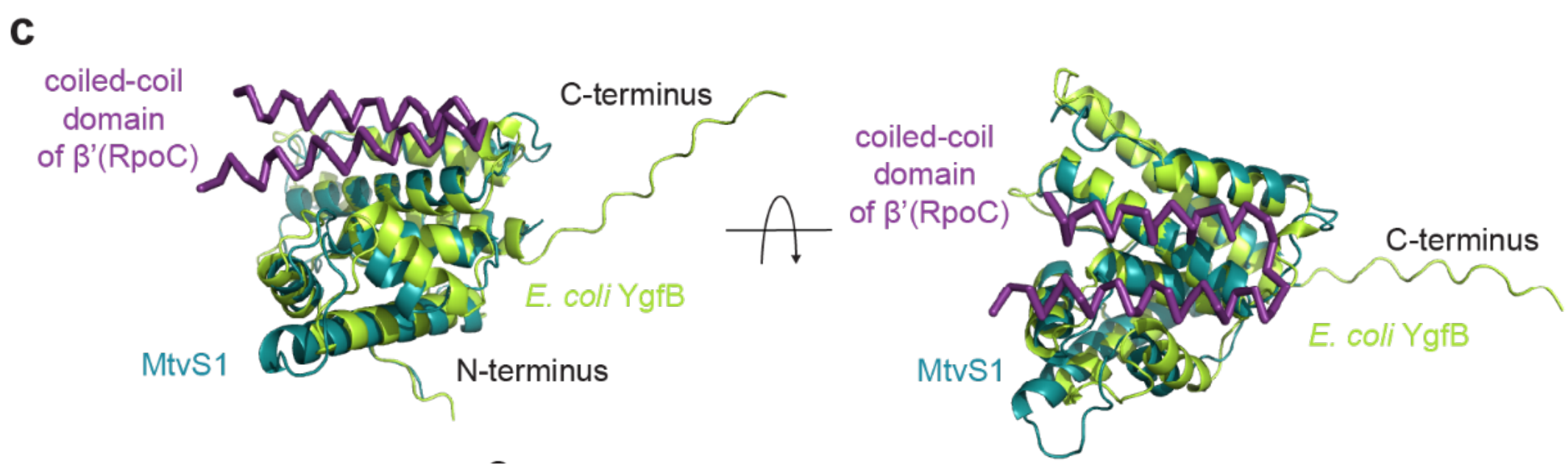

A description of this figure as well as the percentage sequence identity and RMSD values has been added to the text as follows:

"*E. coli* harbors the MtvS1 homolog YgfB (b2909), which is 27.2 % identical at the amino acid sequence level (Fig. 6b) as well as a type I-E CRISPR-Cas system[6] (Extended Data Fig. 6). Structural alignment of the two protein models suggests a highly conserved structure (RSMD value: 1.396 Å over 788 atoms, Fig. 6c, Extended Data Fig. 6a), with many of the MtvS1 residues that are predicted to interact with β' coiled-coil domain conserved in YgfB (Fig. 6b)."

The Extended Data Figure 5 figure legend has some swapped descriptions

Thank you for noticing this mistake. We corrected the figure legends.

Incorrect spelling of locus on line 330

The typo has been corrected.

Figure 4 – presumably should be "raw" not "row"?

The Z-score was calculated on rows and therefore labelled as such in the figure. For clarity, we included a sentence in the figure legend of each heat map:

"Z-scores represent the standard deviation of the actual value from the mean value calculated for each gene across samples."

In addition, we added a description in the methods:

"Z-scores were computed on rows before clustering and describe how many standard deviations the actual value is from the mean value calculated across sample for each gene."

It's surprising that MtvS1 does not bind the cas12a promoter directly, given how it was identified and its effect on cas12a expression. Including the EMSA results—ideally both with and without MtvS2—would be valuable, as this point is central to the mechanism. It's certainly plausible that additional cofactors are required, and identifying such factors would significantly enhance the impact and mechanistic depth of the work.

We agree with the reviewer that it is worth showing the EMSA results. We repeated the experiment with both MtvS1-His$_6$ and MtvS2-His$_6$ individually or combined and found that in contrast to MtvS1-His$_6$, MtvS2-His$_6$ alone is able to bind DNA. Combining MtvS1 and MtvS2 did not alter the results. DNA binding seems to be non-specific as MtvS2-His$_6$ also binds the intragenic control sequence (new Figs. 5a-b):

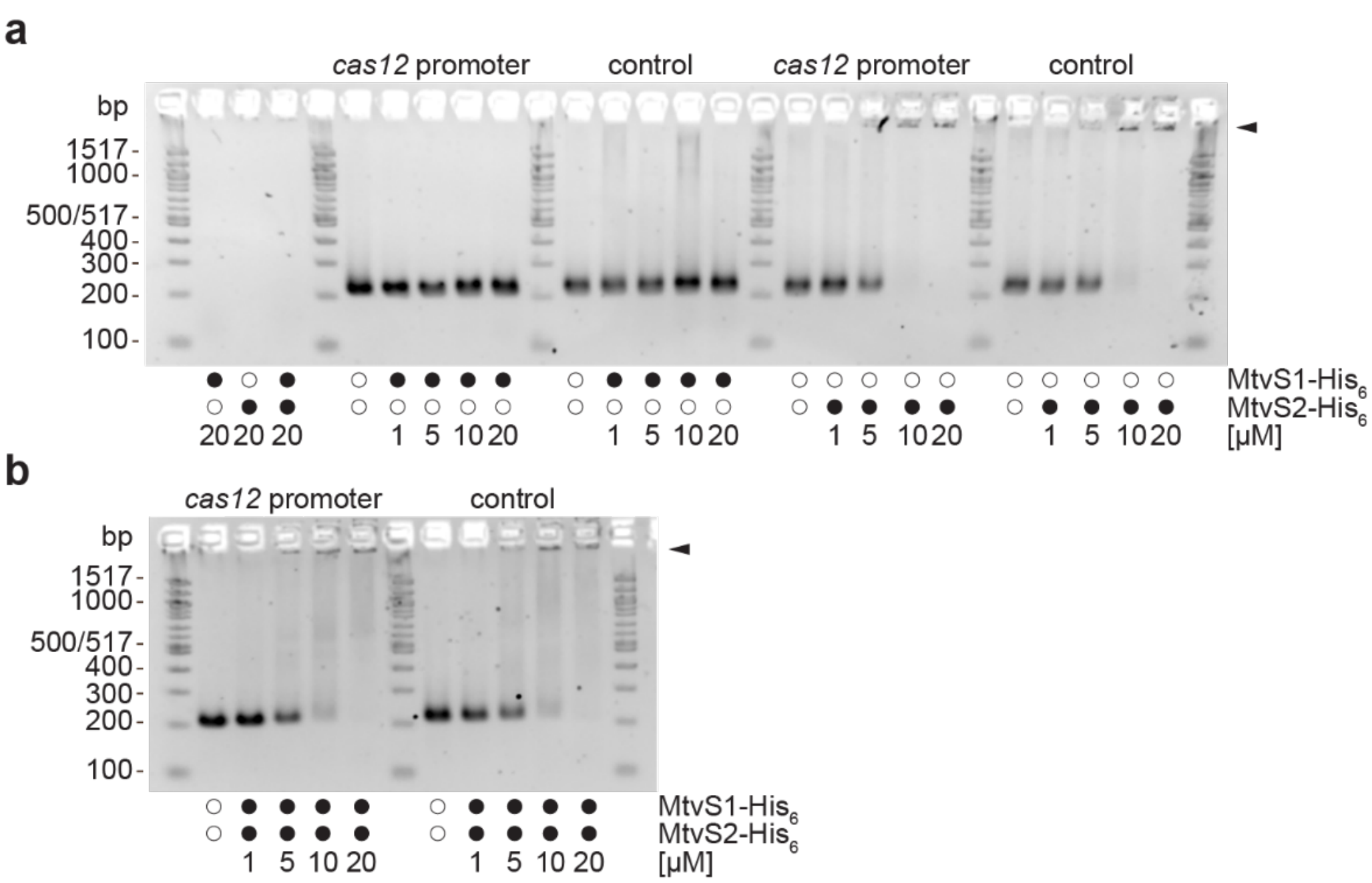

We propose that MtvS1/2 are alternate sigma factors and therefore would require binding to RNAP for specific and high affinity interations. We also agree with the reviewer that additional factors might also be required. Since MtvS2 interacts with RNA polymerase and MtvS1, we speculate that the binding specificity of MtvS2 is determined by either its interaction with RNA polymerase or by MtvS1 binding to RNA polymerase. Alternatively, as the reviewer proposes, there may be additional specificity factors involved. We agree with the reviewer that understanding the molecular mechanism by which these two alternative sigma factors allow promoter selectivity is important, but will require structural studies of RNA polymerase that are currently outside of the scope of this manuscript. We included the new data in the revised text as follows:

> "Electrophoretic mobility shift assays (EMSA) showed binding of MtvS2-His$_6$ to both *cas12* promoter DNA as well as an intragenic *cas12* sequence which we used as a non-specific control sequence (Fig. 5a). On the other hand, we were unable to detect DNA binding for MtvS1-His$_6$ in the tested conditions. Addition of MtvS1-His$_6$ did not alter the DNA shift pattern of MtvS2-His$_6$ (Fig. 5b). Therefore, these results suggest that MtvS1 associates with the *cas12* promoter DNA through interactions with other proteins."

As well as a short discussion of the results:

“Given the non-specific DNA binding of MtvS2, we propose that promoter selectivity and binding are determined either in the complex formed with RNA polymerase or in the presence of additional factors such as MtvS1.”

Reviewer #2 (Remarks to the Author):

The work by Brodmann et al. represents a substantial effort to elucidate the regulation of the Type V-A CRISPR-Cas system in Francisella. The authors report that this CRISPR system is upregulated during stationary phase and under high-cell-density conditions. They identify and characterize two previously unannotated factors, MtvS1 and MtvS2, as responsible for this differential expression. These regulators interact with RNA polymerase subunits, as demonstrated by pull-down assays. This study constitutes a significant and high-quality contribution to understanding CRISPR regulation, an area still underexplored due to the complexity of studying these systems in their native bacterial contexts. Notably, it functionally characterize previously unknown genes that interact with RNA polymerase and may represent noncanonical alternative sigma factors modulating the expression of multiple Francisella genes, including the Type V-A CRISPR-Cas locus. The findings advance our understanding of transcriptional regulation in this opportunistic pathogen.

We thank the reviewer for considering our work "a significant and high-quality contribution" and for valuing our efforts to studying CRISPR immunity in its "native bacterial context".

However, the molecular mechanisms by which MtvS1 and MtvS2 modulate transcription remain uncharacterized.

We agree with the reviewer that understanding the molecular mechanism by which these two alternative sigma factors allow promoter selectivity is important, but will require structural studies of *Francisella* RNA polymerase in complex with these factors and possible DNA as well, that are currently outside of the scope of this manuscript.

Moreover, while the authors suggest an intriguing link between quorum sensing and CRISPR regulation via MtvS1, the evidence provided remains preliminary.

We agree that our initial data was superficial. We have provided more analysis to solidify our claims (see below).

Finally, the study does not directly demonstrate how this regulation impacts CRISPR adaptive immunity against mobile genetic elements, limiting the overall functional conclusions.

We agree with the reviewer. We have obtained evidence that MtvS1/2 can affect anti-plasmid type V-A CRISPR immunity (see below).

<u>Major Comments</u>

1. The main concern is that disruption of mtvS1, despite markedly reducing cas12 transcription, does not impair protospacer interference (Extended Data Fig. 2f). Although cas12 transcript levels decrease, Cas12 protein abundance remains high (Fig. 4c), partially explaining why CRISPR immunity is unaffected. This suggests that MtvS1 and MtvS2 may not be the primary regulators of CRISPR activity, and that additional regulatory factors could be involved.

We agree with the reviewer in that MtvS1-2 dependent regulation of the Type V CRISPR-Cas system is likely not the only regulation of this locus, in particular in other conditions than those we investigated. To highlight the likely possibility of additional regulators we amended our discussion as follows:

> "Regarding the mechanisms behind *cas12* upregulation in stationary phase, we identified MtvS1 and MtvS2, proteins with previously unknown function in *F. novicida* U112, as factors required for the increase in the transcription of the type V-A CRISPR-*cas* locus. While these factors substantially contribute to this increase, anti-plasmid interference assays, fluorescence microscopy visualization of Cas12, and transcriptomics and proteomics data indicated presence of Cas expression in stationary phase even in the absence of MtvS1 and MtvS2. Therefore, we believe it is likely that *F. novicida* encodes additional regulators that feed into the regulatory network for type V-A CRISPR-Cas system activation."

Testing interference efficiency in an mtvS1-S2 double mutant would help determine whether these factors act redundantly or synergistically.

We did test anti-plasmid interference in a conjugation assay using mtvS1/2 double mutant recipients, but failed to detect transconjugants, the same result as in the experiment that tested plasmid transfer into the parental strain. This result is shown in Extended Data Fig. 2n:

n

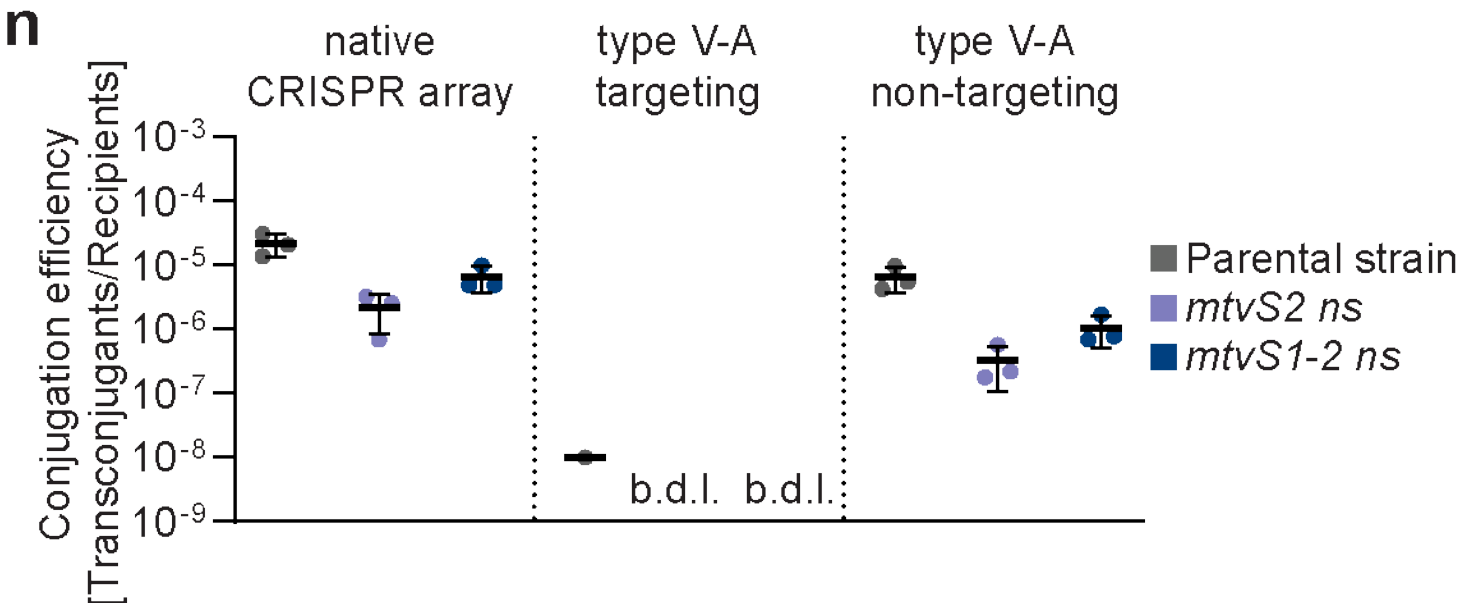

This experiment was briefly mentioned in the text as follows:

> "Plasmid transfer into recipients with *mtvS1* and/or *mtvs2* deletions carrying a targeting spacer was not detected, the same as in the case of conjugation into parental recipients (Extended Data Fig. 2m-n)."

At the same time, the absence of an observable effect on interference may result from the experimental conditions used. The current interference assay evaluate cells during exponential growth, whereas the authors propose that the Type V-A system is upregulated under stationary-phase or high-cell-density conditions to enhance adaptive immunity. Thus, the regulation described here is not directly linked to CRISPR immunity, either at the level of interference or adaptation. To address this, the authors could consider mimicking stationary-phase or high-densitity conditions in their interference assays, for example, by performing experiments in spent overnight medium, under nutrient-limited or minimal-medium conditions, or using alternative delivery systems (e.g., transformation or transduction instead of conjugation). Such approaches would substantially strengthen the biological relevance of the model and clarify whether the observed transcriptional regulation indeed influences CRISPR adaptive immunity.

We agree with the reviewer. Unfortunately, we are not aware of any phage available for infection of *Francisella* species to date. In addition, as explained above, anti-plasmid Cas12 activity is very efficient even in the *mtvS1-2 ns* mutant and the number of surviving transconjugants are below our detection limit. Therefore, we weakened the type V-A CRISPR response to obtain a higher number of transconjugants and look for an observable decrease of this number in the mutant strain. In other words, we extended the dynamic range of the plasmid targeting assay. First, we promoted a general increase in conjugation efficiency by inactivating two restriction-modification systems that limit the establishment of the target plasmid. Second, we introduced into the conjugative plasmid two targets with mutations in their seed sequence. These mutations generate mismatches between the spacer:protospacer sequences that reduce Cas12 DNA cleavage efficiency (PMID 27096362), allowing for an increase in plasmid conjugation. In these conditions, type V-A immunity reduces the conjugation efficiency by an order of magnitude. In the *mtvS1-2 ns* mutant, however, no difference in conjugation efficiency is detected between targeting and non-targeting conditions, a result that demonstrates that these alternative sigma factors are important for immunity. Given the large number of genes that are regulated by MtvS1/2, it is not possible for us to determine if the targeting decrease is only due to lower Cas12 expression in the mutant strain. Indeed, a caveat of this targeting assay is that the conjugation efficiency of the non-target plasmid control is lower in the *mtvS1-2 ns* mutant than in the parental strain, most likely due dysregulation of genes controlled by these factors (we speculate that changes in expression of cell envelope components that we found to be regulated by these alternative sigma factors in our RNA-seq and proteomics results, may be responsible for this general decrease of conjugative transfer in the *mtvS1-2 ns* mutant). These results are shown in the new Fig. 2l:

l

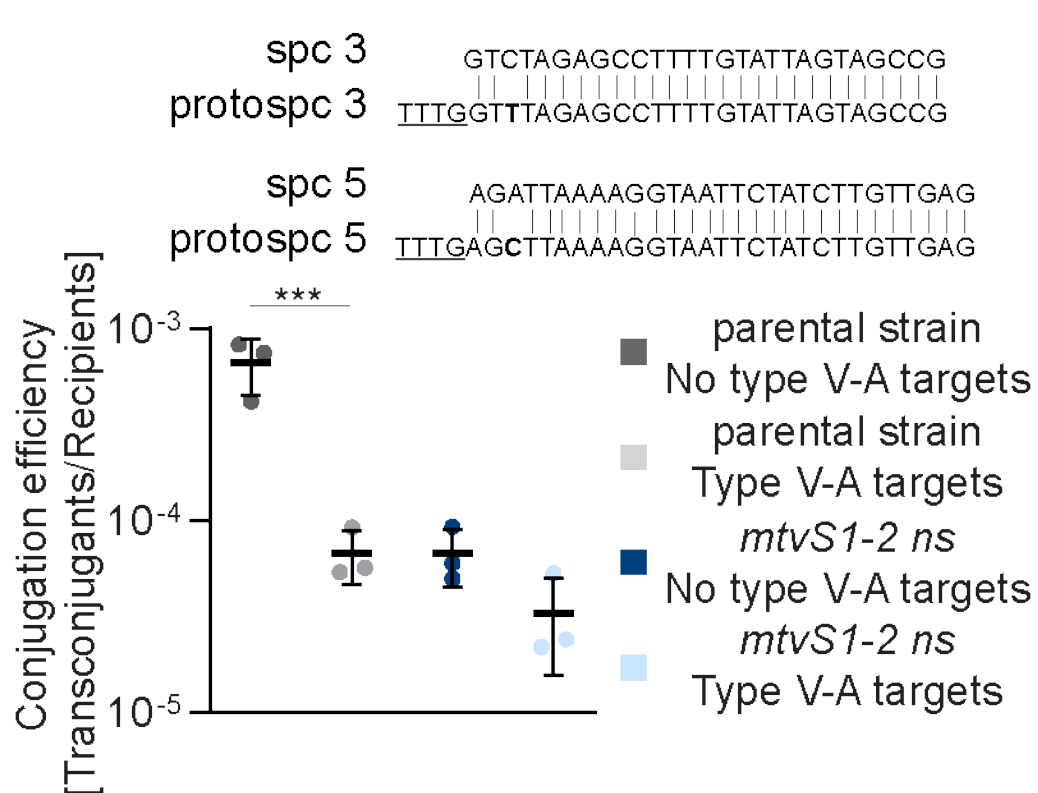

And described in the text as follows:

> "Next, we assessed whether MtvS1-2 dependent regulation of the type V-A CRISPR-Cas locus is important for plasmid restriction. Plasmid transfer into recipients with *mtvS1* and/or *mtvs2* deletions carrying a targeting spacer was not detected, the same as in the case of conjugation into parental recipients (Extended Data Fig. 2m-n). This result prevented us from determining whether the mutations affect plasmid targeting. Therefore, we decided to increase our level of detection of transconjugants to be able to detect differences in anti-plasmid CRISPR immunity, if any. First, we deleted the two restriction enzyme endonucleases, *FTN_0710* and *FTN_1487*, from the parental and mutant strains, which contribute to a low conjugation efficiency. Additionally, we decreased Cas12's targeting activity by introducing mutations in the seed sequence of the protospacer[45]. Since the presence of a single target with a seed sequence mutation completely eliminated immunity, we integrated two protospacers into the conjugative plasmid, matching

spacers 3 and 5 of the native type V-A CRISPR array and containing a mismatch at position 3 within each seed sequence (Fig. 2I). In this condition, the conjugation efficiency of a non-target plasmid into the parental strain was increased from $10^{-5}/10^{-4}$ to ~10^{-3}, and the transfer of a target plasmid produced detectable transconjugants, but with a reduction in conjugation efficiency of one order of magnitude compared to the non-target control (Fig. 2l). In contrast, plasmid targeting did not affect conjugation efficiency into *mtvS1-2 nonsense* mutant recipients (Fig. 2l), a result that indicates that these genes are important for the type V-A CRISPR immune response, presumably due to their effects on *cas12* regulation. Interestingly, conjugation efficiency of the non-target plasmid into *mtvS1-2 nonsense* mutant recipients was reduced by an order of magnitude when compared to the parental strain, a finding that suggests the involvement of these genes in the regulation of other genes outside of the type V-A CRISPR-*cas* operon (see below) that affect conjugation, possibly involved in the generation of cell wall structures."

2. The fluorescence microscopy data in Fig. 3 is central to claim that quorum sensing partecipate to the MtvS1 regulation. This data requires quantification of the fluorescent signal across a large sample size (ideally ~500 cells). As currently presented, the images or videos alone do not convincingly support the claimed reduction, especially given the high background autofluorescence.

We thank the reviewer for pointing this out. In our experiments, there is a single chromosomal copy of the *cas12-mCherry2* gene and therefore the fluorescence signal is low, especially in the microfluidic plates with the autofluorescence of the medium (results were clearer in the absence of autofluorescence when using agarose in PBS pads, in Fig. 1, for example). To clarify our results we now include time-resolved kymographs of the RFP channel, which show that the mCherry2 signal reacted with an increase and then decrease in signal within 1-2 h after media changes (Fig. 4e):

e

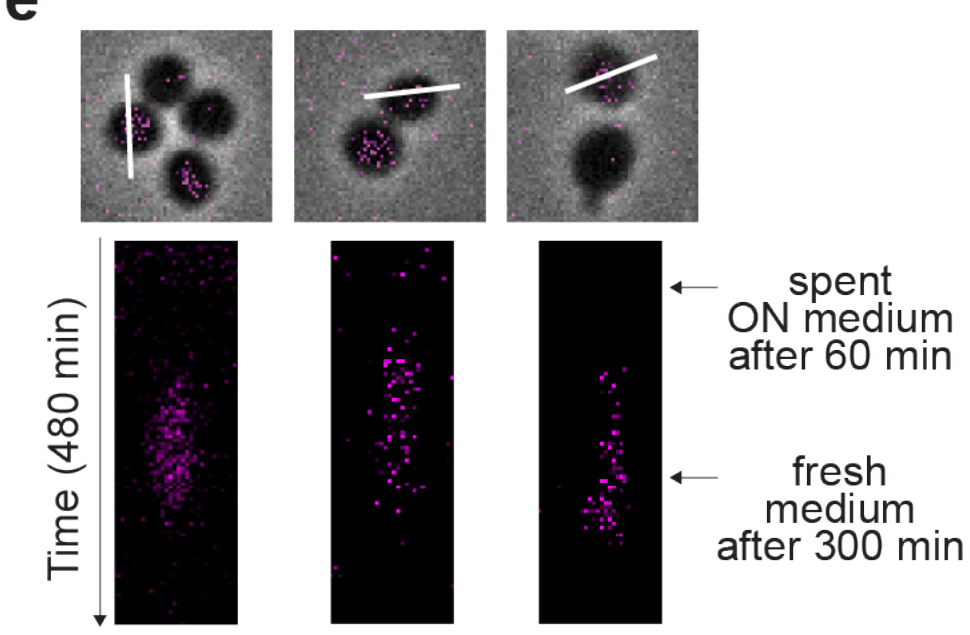

Furthermore, to increase the fluorescence signal in the microfluidic plates, instead of the Cas12-mCherry2 translational fusion we used a transcriptional fusion in which the *cas12* promoter drives expression of mCherry2, cloned on a plasmid to increase the copy number. Higher mCherry2 expression increased the fluorescent signal and highlighted the response to spent medium. These data, along with a kymograph of the RFP channel, are presented in the new Supplementary Video 6 and Extended Data Fig. 4a-c:

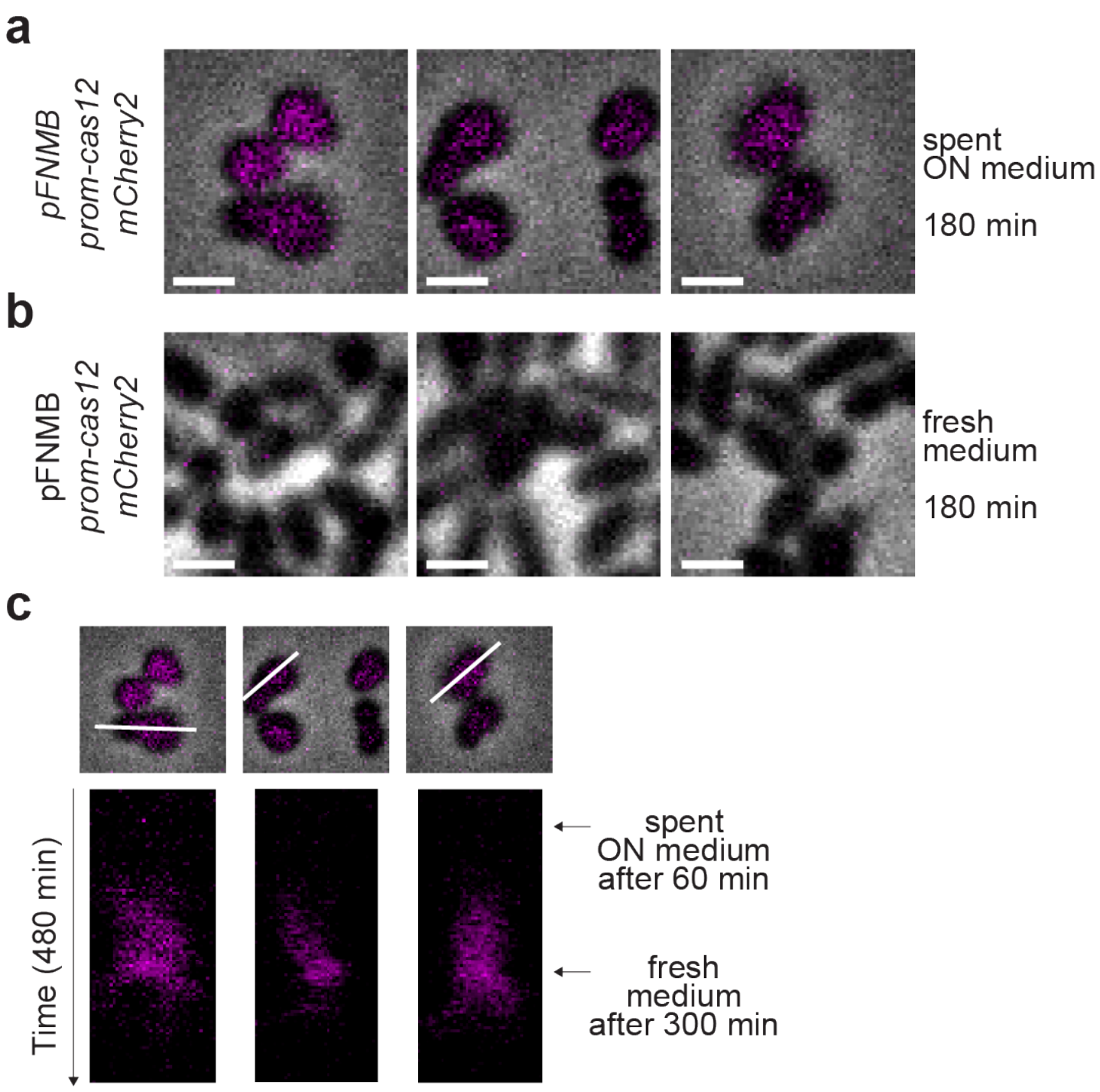

These experiments were described in the revised text as follows:

> "…we tested if the addition of spent overnight medium could increase *cas12* expression in exponential cultures of *F. novicida* U112. We replaced the medium of exponentially growing cells with spent overnight medium (filtered with 0.2 µm filters) in microfluidic plates and monitored individual cells by microscopy. We observed changes in cell shape from rod to coccus, as well as an increase in Cas12-mCherry2 fluorescence (Fig. 4c, Supplementary Video 5). Fresh medium reversed these changes, with cocci converting into rods and resuming growth, and a decrease in Cas12-mCherry2 fluorescence (Fig. 4d). Time-resolved kymographs showed that the mCherry2 signal increased and decreased within 1-2 hours after each media change (Fig. 4e). Since the mCherry2 signal is rather low from the endogenously expressed Cas12-mCherry2, we repeated the experiment expressing mCherry2 under the control of the *cas12* promoter from a plasmid to obtain better visualization of the fluorescence signal than from a single chromosomal locus. Indeed, we detected higher levels of mCherry2 fluorescence, which followed similar dynamics when challenged with spent medium as the endogenously expressed Cas12-mCherry2 (Extended Data Fig. 4a-c, Supplementary Video 6), a result that validated the use of the chromosomally encoded *cas12-mCherry2* strain."

<u>Minor Comments</u>

In general, figures resolutions are not high enough.

Unfortunately, the figures got compressed during uploading. We apologize for the inconvenience; we have uploaded original files in this resubmission to avoid this issue.

Line 47: Citation missing. Please add: Pastuszka, A., Mazzuoli, M.V., Crestani, C. et al. The virulence regulator CovR boosts CRISPR–Cas9 immunity in Group B Streptococcus. Nat Commun 16, 5678 (2025). https://doi.org/10.1038/s41467-025-60871-6.

Thank you for the suggestion. This citation is now included in the introduction.

Line 155: Please discuss the lack of complementation for cas2, the final gene in the operon. More generally, complementation appears less effective for genes further downstream from cas12, could this be due to transcriptional polarity or instability of the downstream transcripts?

We agree with the reviewer's observation and that transcriptional polarity or instability of downstream transcripts could be a cause. If this were the case, restoration of Cas2 transcript levels may require higher levels of MtvS1 than those present in the complemented strain. Since the complementation copy of the gene was inserted in a alternative locus (the wild-type gene is in position 1,307,015 and the second copy is in the Tn7 integration site at position 493,113), different genomic sites could decrease DNA accessibility and thus transcription dynamics and levels of *mtvS1*. We could have tested for transcription differences the parental and complemented strains if we had deleted the *mtvS1* gene, but since we knocked it out through the introduction of non-sense mutations the complemented mutant produces a mixture of wild type and mutated *mtvS1* mRNAs. In contrast, *mtvS2* complementation, which was performed using a plasmid-borne copy of the gene that should lead to its over-expression, produced a complete restoration of the qRT-PCR values, a result that supports the above hypothesis for lack of rescue of cas2 RNA levels in the *mtvS1*-complemented strain. We modified the manuscript to mention this analysis:

> "We restored MtvS1 expression in the *mtvS1 nonsense* mutant by integrating a copy of the gene, including its promoter sequence, at the Tn7 integration site downstream of gene *glmS* (*FTN_0485*). With the exception of *cas2*, the transcripts for all the *cas* operon genes were increased (Fig. 2c-f). A possible explanation for lack of restoration of *cas2* transcription is the level of expression of MtvS1 from its alternative chromosomal location in the complemented strain, which could affect the DNA accessibility and transcription dynamics of the integrated copy of *mtvS1*, and thus lower the expression of this gene. If this were the case, given that *cas2* is the last gene of the operon and could display low RNA levels due to transcription polarity, or if the *cas2* transcript is unstable, rescue of the RT-qPCR values could require a higher concentration of MtvS1 that the present in the complemented strain. Expression of MtvS2 was restored with a plasmid-borne copy of the mtvS2 gene that also included its promoter, which was sufficient to increase the RT-qPCR values of all *cas* genes in the operon to parental levels (Fig. 2g-j)."

Figure 1b/c: Dotted lines are not visible.

Thank you for pointing this out. We fixed as follows it for all graphs:

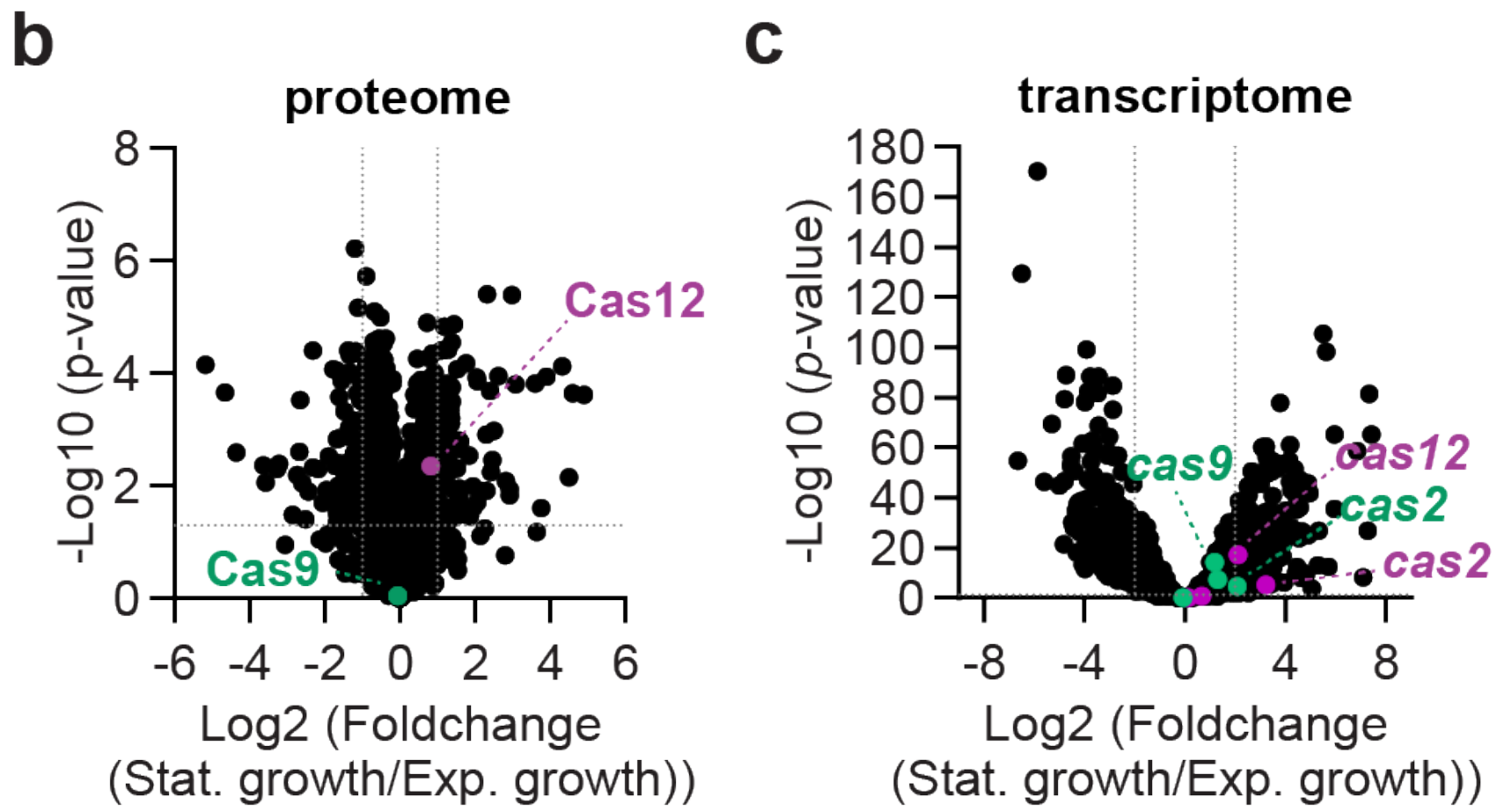

Extended Data Fig. 2h: While mtvS1 transcript and protein levels do not vary between exponential and stationary phases, the mtvS1-mCherry fusion shows increased protein levels in stationary phase. Could this reflect enhanced stability of the tagged variant? Please comment.

We agree with the reviewer with this possibility. However, it is difficult to extrapolate from this result about the stability of MtvS1, since fluorescent tags can alter behavior of proteins, including aggregation and stability (PMIDs 22289035, 28241009, 22484850). We mention this possibility in the revised text as follows:

> "Fluorescence microscopy suggests the possibility higher proteins level for MtvS1-mCherry2 in stationary phase, however no increase of protein levels was observed with LC-MS/MS (Fig. 2l, Supplementary Data File, tab A). It is possible that the fluorescence tag affected MtvS1 aggregation and/or stability properties[61-63]."

Figure 4 legend: Please specify what the Z-score reflects in the legend.

Thank you for pointing out the lack of description. We included now a sentence in the figure legend of each heat map:

> "Z-scores represent the standard deviation of the actual value from the mean value calculated for each gene across samples."

In addition, we added a description in the methods:

> "Z-scores were computed on rows before clustering and describe how many standard deviations the actual value is from the mean value calculated across sample for each gene."

Lines 214-222: The promoter analysis is not clearly linked to the conclusions. The presence of canonical motifs (TATAAT at –10, a variable –35 region) in both upregulated and downregulated promoters does not seem to distinguish classes of promoters or inform the regulatory mechanism. Please clarify the rationale and implications.

We agree with the lack of clarity on this point. This promoter analysis was an attempt to find a putative -35 sequence that is characteristic for MtvS1-2 dependent promoters. Our RNA-seq experiments do not allow a distinction between direct targets of MtvS1-2 dependent regulation and genes that are up or down regulated as compensation for the nonsense mutations introduced in the alternative sigma factors. Therefore, we first filtered for promoters that have a canonical -10 sequence (TATAAT), i.e., similar to the *cas12* promoter used to identify MtvS1-2. Since many promoters contain a canonical -10 sequence, it was encouraging that the TATAAT sequence was enriched in promoters of down regulated genes compared to promoters of up regulated genes in the mutants, strengthening the hypothesis that this sequence is important for MtvS1-2 dependent regulation. To clarify this point we have now included the Gene Set Enrichment Analysis (GSEA) data of both mutants (Extended Data Figure 3c):

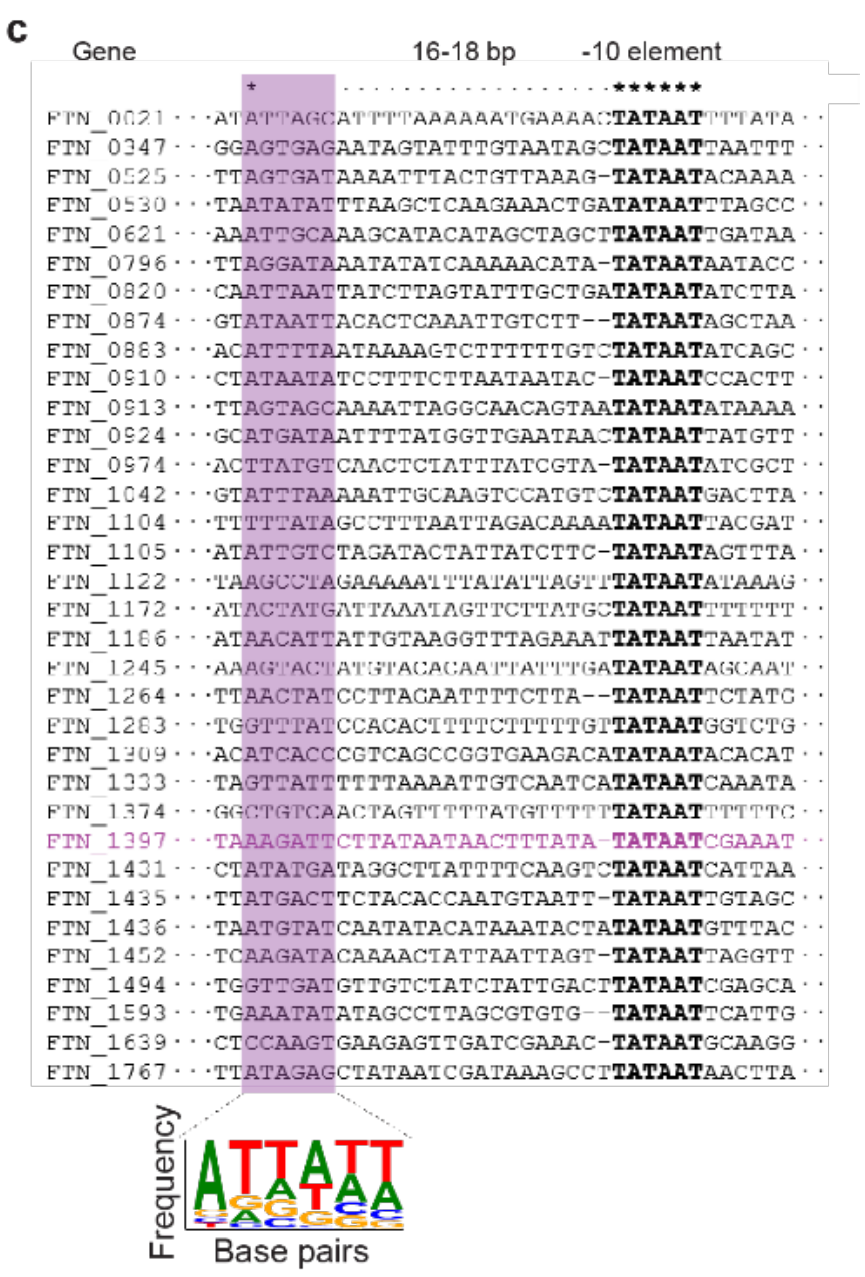

We also rewrote this section in the revised manuscript as follows:

> "To find the putative -35 sequence associated with MtvS1-2 regulation, we analyzed the promoter sequences of the operons that were significantly down-regulated in stationary phase in either of the two mutants, compared to the parental strain (Supplementary Data File, tab E and H). Given that some of these genes may not be directly regulated by these factors (their expression could be a response to change in other factors that are directly regulated by MtvS1/2), we decided to filter the promoter sequences for the presence of the canonical -10 sequence (TATAAT), which is present in the *cas12* promoter used as bait to identify MtvS1 and MtvS2 (Fig. 2b, Supplementary Data File, tab J). We found that 61 % of the promoters of down-regulated genes (34/56) encoded a TATAAT sequence. In contrast, only 35 % of promoters (14/37) of up-regulated operons in stationary phase contained the canonical -10 element (Supplementary Data File, tab E and H). We believe that this enrichment is in agreement with the hypothesis that the canonical -10 sequence is important for MtvS1-2 dependent gene expression. Finally, we aligned the filtered promoters and detected a putative -35 sequence starting with a highly conserved A (Extended Data Fig. 3c)."

Line 229: Correct reference to Fig. 4d instead of Fig. 4e.

Thank you for noticing. We corrected the mistake.

Line 225: Correct reference to Fig. 4c instead of Fig. 5d.

Thank you for noticing. We corrected the mistake.

Lines 234-238: The rationale for highlighting specific genes is unclear. Please explain the selection criteria and how these examples support your conclusions.

We agree with the reviewer. We rewrote this section entirely to focus on the GSE analysis for clarity:

> "Other, statistically significant, changes in protein levels were observed in the absence of MtvS1 (Fig. 3e and g, Extended Data Fig. 3d). The GSE analysis suggests an upregulation of the UMP biosynthesis proteins in exponential growth phase in the *mtvS1* mutant, a result that suggests that MtvS1 represses this pathway. In stationary growth phase, in the absence of MtvS1 the levels of proteins participating in bioenergetic processes were significantly decreased and some cell envelope proteins were more abundant, compared to the parental strain. (Supplementary Data File, tab B)."

Lines 253-254: A standard western blot cannot exclude post-translational modifications such as phosphorylation. Please rephrase accordingly.

We respectfully disagree with the reviewer, since it it well-evidenced that phosphorylation can alter the mobility of proteins on SDS-PAGE (PMIDs 12794087, 30706340). Moreover, we believe that we used cautious language to conclude about the result, stating that it only "suggests" (does not prove) the lack of "major" modifications (cannot exclude the presence of minimal modifications that do not alter protein mobility). To clarify this issue, we modified the text as follows:

> "We performed western blot analysis of MtvS1-His_6 expressed in *Francisella* to look for changes in electrophoretic mobility in stationary phase. Such changes could indicate amino acid modifications not present in the heterologously expressed protein that may affect its DNA binding activity. The mobility of MtvS1-His_6, however, did not change in the different growth phases (Extended Data Fig. 5e), a result that suggests lack of major protein modifications."

Line 364: The idea that Francisella RNA polymerase may be required for in vitro transcription is intriguing. Have the authors compared the Francisella and E. coli polymerase subunit compositions or sequence divergence to support this hypothesis?

We thank the reviewer for raising this point that needs clarification. Several structural analyses of *Francisella* RNA polymerase revealed the presence of two distinct α-subunits that form a heterodimer in the core enzyme ($\alpha^1\alpha^2\beta\beta'$) (PMIDs 33217319, 17571921, 22108176). These are evolutionarily distinct from the α-subunits of other γ-proteobacteria, including *E. coli*, which encodes an α-subunit homodimer in the core enzyme ($\alpha_2\beta\beta'$). We believe that it is possible that these substantial differences could play a role in our *in vitro* transcription assay, and therefore have modified the text to include this explanation as follows:

> "Alternatively, *in vitro* transcription assays could have failed due to the need of *Francisella*, and not *E. coli*, RNA polymerase. *Francisella* encodes two different α-subunits that form an $\alpha^1\alpha^2\beta\beta'$ core enzyme[54,55] and are evolutionarily distinct from other γ-proteobacteria, including *E. coli*, that harbor a single α-subunit gene

and form an $\alpha_2\beta\beta'$ core enzyme. Since α C-terminal domains can interact with promoter DNA elements, it is possible that E. coli RNAP cannot appropriately recognize the *cas12* promoter.”

Reviewer #3 (Remarks to the Author):

The study by Brodmann and colleagues reports the regulation of CRISPR-Cas expression in Francisella tularensis. The strain under study contains two CRISPR-Cas systems, of types II-B and V-A, respectively. The authors show that the type II-B system is constitutively expressed, while the type V-A system is induced during stationary phase and at high cell density. The experimental data clearly support these observations, which are in agreement with previous data indicating the upregulation of defence mechanisms in stationary phage, as the authors also indicate (line 319).

In the study of the mechanisms involved in the regulation of the type V-A system, the authors identified two proteins, MtvS1 and MtvS2, and clearly showed that they play a role in transcriptional regulation of that system. This finding is of particular interest because the characterization of proteins related to MtvS1 remains very limited, and no proteins similar to MtvS2 have been characterized to date. The authors have performed a broad range of well-designed experiments using different complementary techniques. As a result, the authors propose that these proteins act as alternative sigma factors. This is an attractive hypothesis supported by some evidences.

We thank the reviewer for considering that our results “clearly support” the existence of regulation of the type V-A CRISPR-*cas* locus and that our work presents “a broad range of well-designed experiments”.

However, the data provided are not conclusive. In fact, in vitro transcription assays using the E. coli RNA polymerase holoenzyme failed to reconstitute the transcriptional activity. As the authors indicate in the manuscript, different results might have been obtained using the Francisella RNA polymerase, but those experiments have not been reported.

We acknowledge the reviewer’s disappointment. We think that to fully understand the molecular mechanism by which these two alternative sigma factors allow promoter selectivity, it will be required not only to perform in vitro studies, but also structural studies, of *Francisella* RNA polymerase in complex with these factors and possible DNA as well. We believe that such experiments will take years to complete and therefore are outside of the scope of this manuscript.

Specific comments.

1. The introduction of the manuscript would have been improved by covering the regulation of CRISPR-Cas systems in a more detailed way. In line 44, the authors mention the differential regulation of coexisting CRISPR-Cas systems and cite reference 14, which does not address this topic. Moreover, listing 21 references in a single sentence (line 47) is not very useful to put into context the study submitted.

We thank the reviewer for correcting our references. We have replaced former reference #14 which, as the reviewer indicates, does not describe regulation nor the presence of co-existing CRISPR loci, for PMID 31745554. This paper states that 23 % of all genomes containing a CRISPR-Cas system encode more than one system and

therefore is appropriate to support this claim. In addition, we have expanded the introduction about CRISPR-Cas system regulation for better overview and context. With this change, the 21 references are now separated as specific citations:

> "Regulation of the CRISPR-Cas defense is important to balance an effective immune response that minimizes autoimmunity and limits, but does not completely abrogates, potentially beneficial mobile genetic elements[1,15–18]. Previous studies have shown that regulation of the CRISPR-Cas systems can be affected by secondary metabolites and nanoRNAs[19–24], H-NS-mediated chromosome condensation and membrane stress[19,20,25–27], environmental cues sensed by two component signaling systems[28,29] and cell density or virulence[30-33]. Many CRISPR-Cas systems can be autoregulated by repression of their own transcription through the Cas effectors[34–38]. Other CRISPR-Cas systems encode their own specific transcriptional regulator or putative DNA-binding proteins within or nearby the CRISPR-*cas* locus[34,39–43]. Detailed investigation of regulatory networks governing CRISPR expression, however, has been difficult due to the lack of natural hosts that can be used as experimental systems to study the CRISPR-Cas response."

2. The quality of several figures in the manuscript should be improved. Some are difficult to interpret clearly. For example, Figures 1b (adding arrows to identify the proteins would help), 1c, 2a, 2b, etc. The figures appear blurred which I wonder if it could be due to file compression during the submission process.

We apologize for the bad resolution, which was indeed a consequence of image compression during submission. We now optimized the PDF conversion to avoid resolution loss.

To clarify the volcano plots, we added lines identifying the proteins/genes. We show below Fig. 1b as a representative example of the new version of these figures:

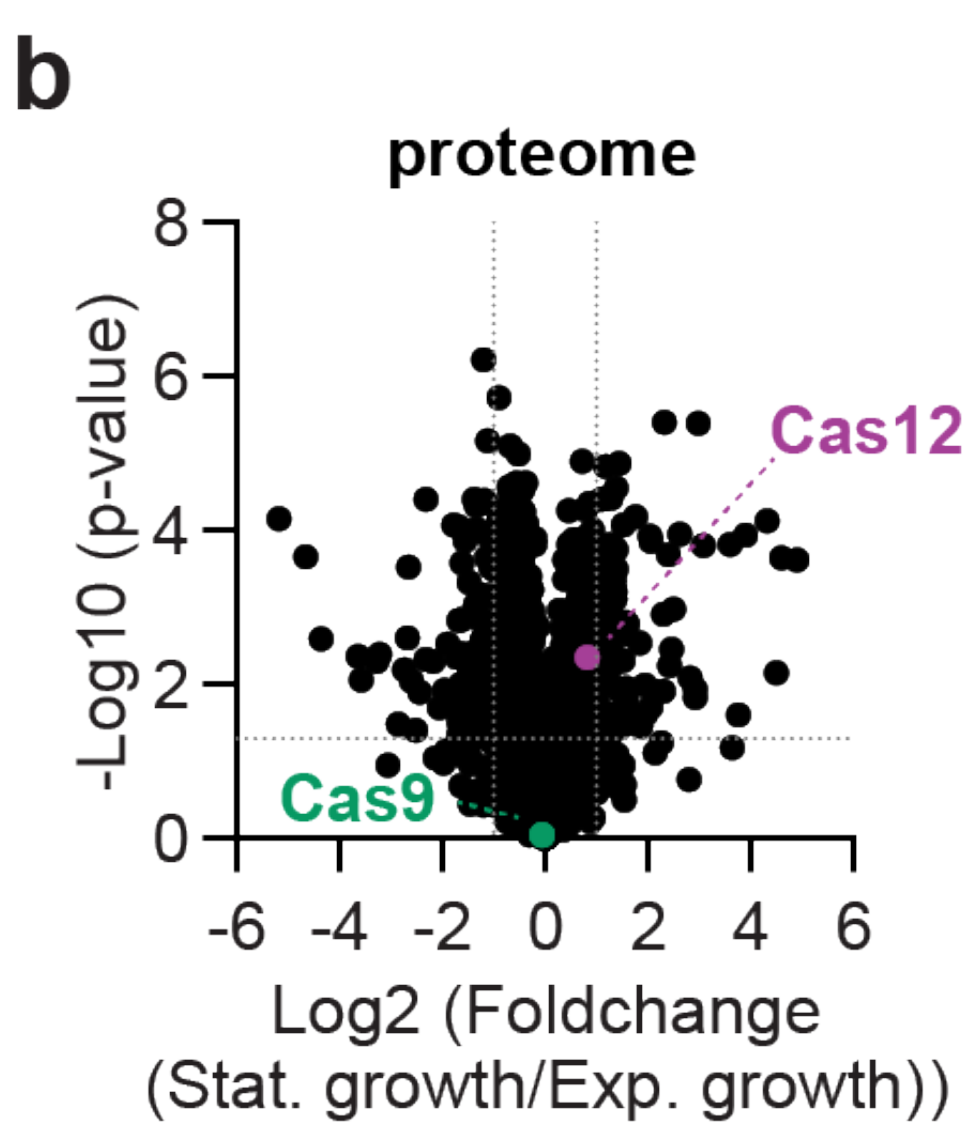

3. Figure 1 legend: It is mentioned that "Dotted horizontal line: p-value of 0.05. Dotted vertical lines: 2-fold difference." However, those dotted lines are not shown in the figure.

Thank you for catching this mistake. We have added the missing lines into all graphs. We show below Fig. 1c as a representative example of the new version of these figures:

c

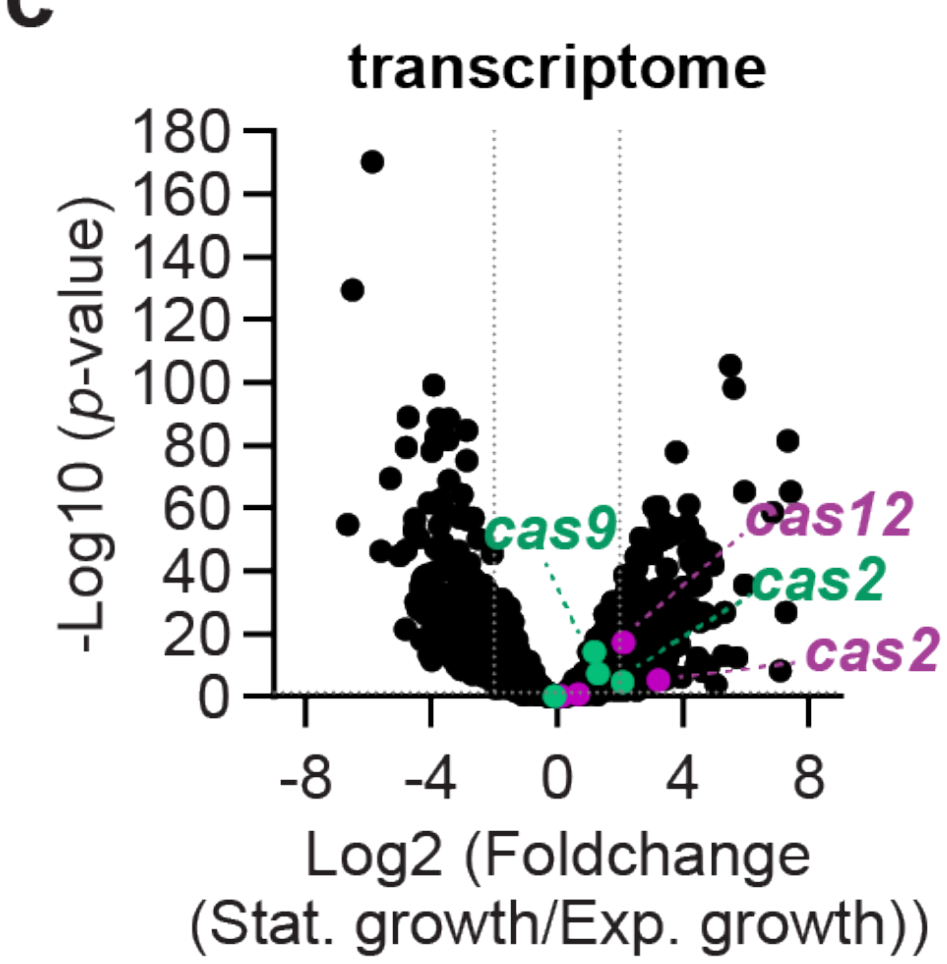

4. Line 114: the authors refer to the "small repeat unit (SRU)" while extended data figure 1B used the term "single repeat unit". It is also unclear whether the SRU could have been represented in Fig. 1g. It would have been informative to indicate where SRU is located in relation to the -35 and -10 sites.

Thank you for noticing this inconsistency. We now only use the term "single repeat unit", as initially defined by Shmakov *et al*. (PMID 37090614). The predicted -35 site lies within the putative SRU, which is now highlighted in grey in Fig. 1g:

Figure 1g:

g

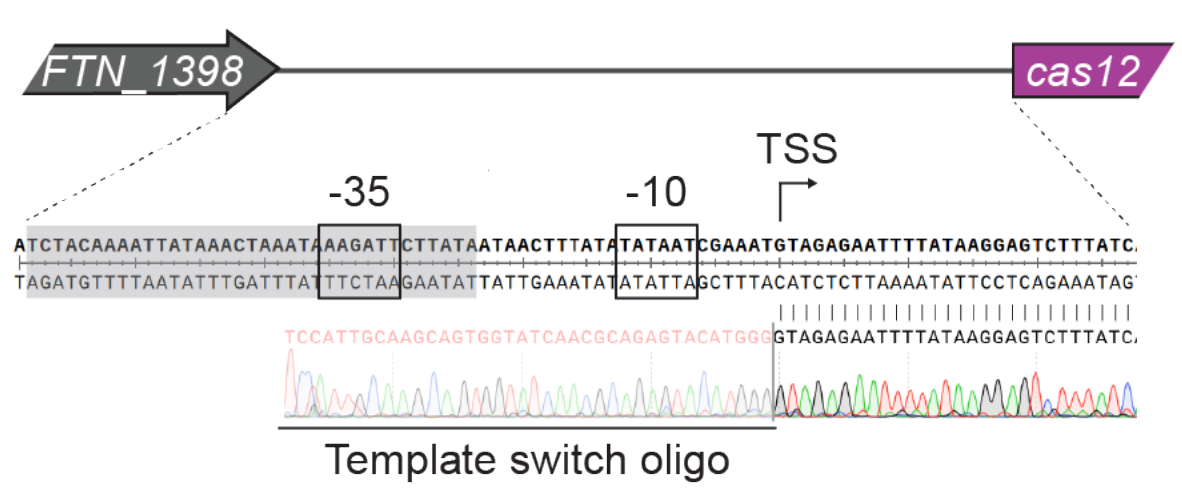

We also clarified the text as follows:

> "The transcription start site map via 5' RACE experiments suggested the presence of a putative promoter sequence with a canonical -10 sequence (TATAAT), but a divergent -35 sequence (AAGATT, canonical sequence: TTGACA) that lies within the putative SRU (Fig. 1g)."

5. Line 117: the authors mention that "the fluorescence pattern did not change in absence of cas12 (Fig. 1f)". It would be useful to clarify that these results should be compared with those in Fig. 1a (lower pannels).

We agree. We re-wrote the sentence as follows:

> "However, the fluorescence pattern observed in Fig. 1a (lower panel) did not change in absence of *cas12* (Fig. 1f)."

6. Lines 123-124: the phrase would improve by indicating clearly that the sequence AAGATT shown is the one detected in the cas12 promoter, and by including the canonical sequence for comparison.

We agree with the reviewer. We now include the canonical -35 sequence for comparison:

> "The transcription start site map via 5' RACE experiments suggested the presence of a putative promoter sequence with a canonical -10 sequence (TATAAT), but a divergent -35 sequence (AAGATT, canonical sequence: TTGACA) that lies within the putative SRU (Fig. 1g)."

7. The sentences in lines 223-225 are confusing. Please indicate where the data for Cas12 protein levels are shown. The mention of fig. 5d is not correct, if the comparison is at the proteomic level, it seems that Fig, 4d should mentioned.

Thank you for pointing this out. We rewrote this paragraph as follows:

> "Last, to corroborate the observed changes in transcription observed in the absence of MtvS1, we performed proteomic analysis of the parental and *mtvS1 nonsense* mutant strains in both growth phases. The data confirmed the increase in Cas12 protein levels when cells enter stationary phase, which was decreased in the absence of MtvS1 (Fig. 1b and 3e, Extended Data Fig. 3d, Supplementary Data File, tab A). Although the decrease of Cas12 levels in the mutant strain is not statistically significant, this result is consistent with the decrease in the Cas12-mCherry2 fluorescence observed using microscopy (Fig. 3f). In spite of this decrease, microscopy showed residual fluorescence signal in the *mtvS1 nonsense* mutant, which was also confirmed by whole-cell proteomics results that revealed a small but detectable increase of Cas12 protein levels in stationary phase compared to exponential growth in this mutant (Fig. 3e-f, Extended Data Fig. 3d, Supplementary Data File, tab A). This result provides a possible explanation for the high Cas12-dependent plasmid targeting levels detected for the *mtvS1* nonsense mutant strain (Extended Data Fig. 2m)."

8. Line 227h the correct figure reference should be Extended data Fig. 2f, not 2b.

We corrected this mistake.

9. Line 229: Fig. 4e is mentioned, but this figure doesn't exist (perhaps it should be Fig. 4d).

It is indeed Fig. 4d the one that should have been referenced. We corrected this mistake.

10. Extended Fig. 5. Please check the legend, as some figure panel references are incorrect. For example, legend "a" refers to panel "B".

Thank you for noticing. We corrected the figure legends.

11. Sometimes, the data labeling could be improved. For example, in line 138 it is indicated that FTN_1238 as enriched (Fig. 2b), yet the figure labels it as Mtvs1, a

designation first introduced in line 165. Similarly, in lines 293-295 (Fig. 6a), FTN_1519 is mentioned, while the figure uses the MtvS2 name, first introduced in line 296.

We have put the new names of these genes in parenthesis the first time we introduce *FTN_1238* (*mtvS1*) and *FTN_1519* (*mtvS2*) to match the figures:

> "On the other hand, in the stationary growth sample, FTN_1238 and FTN_1519, which we renamed *mtvS1* and *mtvS2* respectively (see below), were significantly enriched (Fig. 2b, Supplementary Data File, tab J)."

12. Line 666: change "transformants" for "transconjugants".

Thank you for pointing out this mistake, which is now corrected.

Reviewer #4 (Remarks to the Author):

I co-reviewed this manuscript with one of the reviewers who provided the listed reports. This is part of the Nature Communications initiative to facilitate training in peer review and to provide appropriate recognition for Early Career Researchers who co-review manuscripts.

Thank you for your review.

Reviewer #1 (Remarks to the Author):

In this resubmitted manuscript, Brodmann, et al include valuable new data that explores the functional impact and broader relevance of two proteins, MtvS1 and MtvS2, on CRISPR-Cas expression. Namely, they show that homologs of MtvS1 are widespread in bacteria and impact expression of type I-E CRISPR-Cas systems, supporting a broader role in CRISPR-Cas regulation. They also provide evidence that mutations in MtvS1/2 decrease Cas12a-mediated targeting of plasmids compared to control (albeit with some inherent confounding factors, which the authors explicitly describe). These additions show a broader relevance, which ameliorates some of my concerns. The authors also provide additional data on MtvS2.

The claims are supported by the evidence and the methodologies appear sound. The main issue, raised by myself and other reviewers, is that the impact on CRISPR-Cas expression is mild and the functional impacts of MtvS1 and MtvS2 remain unclear. The data provided in figure 2I provide some support for impact but rely on a compromised Cas12a system and with confounding factors that cannot be avoided. The authors have clearly done a lot of work that is valuable to the field, but it still remains unclear to me how important these transcriptional regulators are for CRISPR-Cas biology, per se, which reduces the significance of the work. Given that E. coli has many phages, it would be interesting to see if YgfB impacts phage targeting by type I-E CRISPR-Cas, given that Francisella lacks known phages. All the same, the characterization of two widespread transcriptional regulators that have broad impacts on bacterial gene expression is a notable contribution to bacteriology, even if the relative impact on CRISPR-Cas expression is limited.

We thank the reviewer for the positive comments. Future work in both E. coli and Francisella will certainly address the impact of these transcription regulators beyond CRISPR immunity.

Reviewer #2 (Remarks to the Author):

The revisions made by the authors are very satisfactory, and I believe they have significantly improved the quality of the manuscript. My main concern in the previous version was the lack of an observable effect on Type V-A interference in the mtvS1-2 mutant. However, despite the limitations of the available tools, the authors developed a very clever strategy to increase the dynamic range of the plasmid-targeting assay. This substantially strengthened the experimental evidence.

The other points I raised, some of which were also noted by the other reviewers, have been adequately addressed. In particular, the demonstration of the conservation and activity of MtvS1 homologues in E. coli broadens the relevance of the findings and increases the overall impact of the study.

I strongly support publication of this work in your journal. The regulation of CRISPR systems remains an important and underexplored area, despite the widespread use and application of CRISPR technologies, in large part because these systems are experimentally challenging to study. The methodology used in this manuscript meets high standards in the field, and the experimental work is comprehensive, rigorous, and sound.

We thank the reviewer for appreciating the work we put in performing the revisions and the positive comments.
