# Supplementary material for: MtvS1 and MtvS2 Interact with RNA polymerase to regulate the Francisella type V-A CRISPR-Cas system
Source: Nat Commun. 2026 Sep 1;17:10400. doi: 10.1038/s41467-026-77062-6 (PMC13627669; doi:10.1038/s41467-026-77062-6)
Supplement: Supplementary file 3 [file 41467_2026_77062_MOESM1_ESM.pdf]

Supplementary Figures 1-6

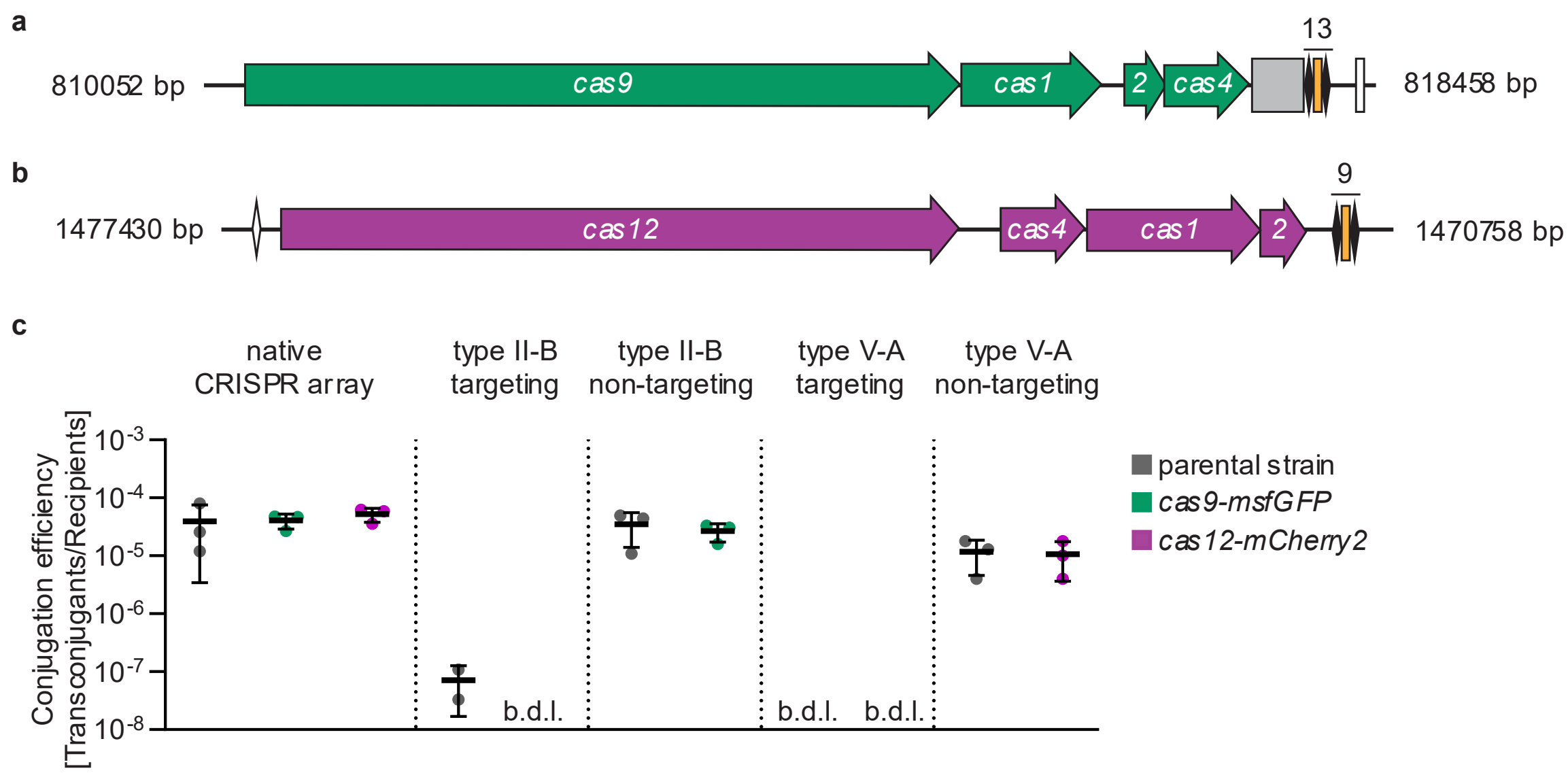

Supplementary Figure 1

C-terminal fluorescent tags do not inhibit Cas9- and Cas12-mediated plasmid targeting.

a Type II-B CRISPR-*cas* system in *F. novicida U112*. Green arrows: *cas* genes. Grey box: *leader/tracr sequence*. Black diamonds: *repeats*. Orange box: *spacers*. White box: *scaRNA*. Drawn in scale.

b Type V-A CRISPR-*cas* system in *F. novicida U112*. White diamond: *single repeat unit*. Magenta arrows: *cas* genes. Black diamonds: *repeats*. Orange box: *spacers*. Drawn in scale.

c Assessment of interference capability of wild-type and C-terminal tagged Cas9 and Cas12 in a conjugation-based plasmid inhibition assay. Strains have both native type II-B and type V-A CRISPR arrays or have the respective CRISPR array replaced by a spacer matching a protospacer on the conjugated plasmid (targeting spacer) or one that does not match (non-targeting spacer). Grey circles: parental strain. Green circles: *cas9-msfGFP*. Magenta circles: *cas12-mCherry2*. b.d.l.: below detection limit. Means with standard deviations are displayed (*n* = 3 biological replicates). Source data are provided as Source Data file.

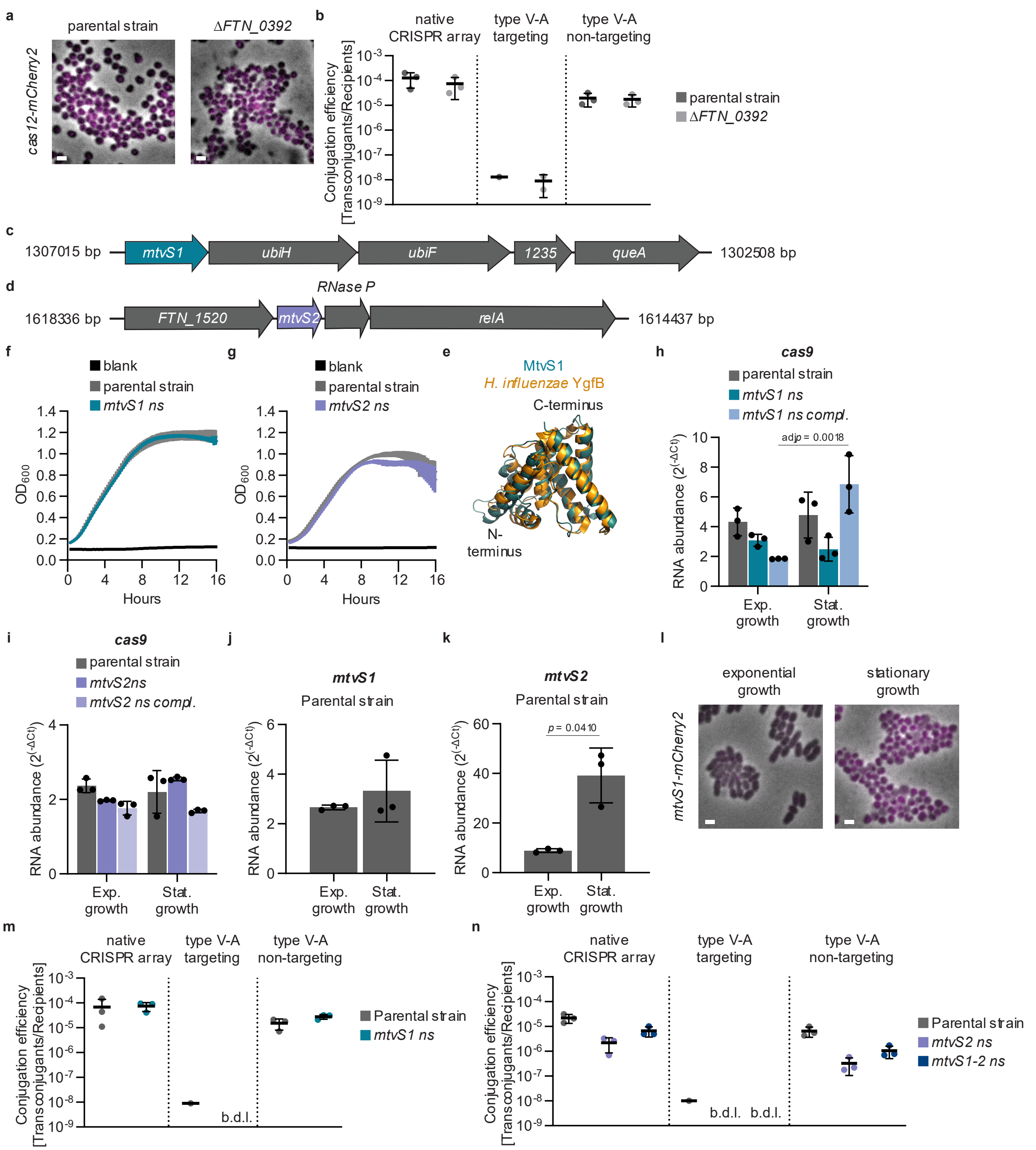

Supplementary Figure 2

MtvS1 and MtvS2 have different expression patterns.

a Fluorescence microscopy of Cas12-mCherry2 in parental strain and *ΔFTN_0392* mutant. Comparable fluorescent intensities in stationary growth were detected. Merge of phase contrast and RFP channel in 13 x 13 µm fields of view are shown. Scale bars represent 1 µm. Representative images are shown (*n* = 3 biological replicates). Source data are provided as Source Data file.

b Assessment of interference capability of parental strain and *ΔFTN_0392* mutant in a conjugation-based plasmid inhibition assay. Strains have a native type V-A CRISPR array or have a native type V-A CRISPR array replaced by a spacer matching a protospacer on the conjugated plasmid (targeting spacer) or one that does not match (non-targeting spacer). Grey circles: parental strain. Purple circles: *ΔFTN_0392* mutant. Means with standard deviations are displayed (*n* = 3 biological replicates). Source data are provided as Source Data file.
c *mtvS1* is the first gene of a conserved operon which also includes *ubiH* (2-octaprenyl-6-methoxyphenyl hydroxylase), *ubiF* (2-octaprenyl-3-methyl-6-methoxy-1,4-benzoquinol hydroxylase), *FTN_1235* (putative phage protein) and *queA* (S-adenosylmethionine:tRNA ribosyltransferase-isomerase).
d *mtvS2* is the second gene of a predicted operon which also includes *FTN_1520* (amino acid transporter), *RNAse P* RNA and *relA* (GTP pyrophosphokinase).
e Comparison of AlphaFold 3 MtvS1 structure (turquoise) with solved *H. influenzae* YgfB structure (yellow, 1IZM). RSMD value: 1.258 Å over 765 atoms.
f-g Growth curves of parental strain and *mtvS1 nonsense* **(f)** or *mtvS2 nonsense* **(g)** mutants. OD600 was measured every 10 min. Black: blank. Grey: parental strain. Turquoise: *mtvs1 nonsense* mutant. Purple: *mtvS2 nonsense* mutant. Means with standard deviations are displayed (*n* = 3 biological replicates with each 3 technical replicates). Source data are provided as Source Data file.
h-i Determination of mRNA levels for *cas9* in the *mtvS1 nonsense* **(h)** and *mtvS2 nonsense* **(i)** mutants by RT-qPCR. Grey bars: parental strain (with empty plasmid for **i**). Turquoise bars: *mtvS1 nonsense* mutant. Light blue bars: *mtvS1 nonsense* mutant with wild-type *mtvS1* at Tn7 insertion site. Purple bars: *mtvS2 nonsense* mutant with empty plasmid. Light purple bars: *mtvS2 nonsense* mutant with wild-type *mtvS2* expressed from plasmid. Means with standard deviations are displayed (*n* = 3 biological replicates). Significant differences were determined with a two-way ANOVA and Tukey's multiple comparison test (95 % confidence interval). Source data are provided as Source Data file.
j-k Determination of mRNA levels for *mtvS1* **(j)** and *mtvS2* **(k)** in the parental strain by RT-qPCR. Grey bars: parental strain. Means with standard deviations are displayed (*n* = 3 biological replicates). Significant differences were determined with Welch's *t*-test (95 % confidence interval). Source data are provided as Source Data file.
l Fluorescence microscopy of MtvS1-mCherry2 in parental strain. Fluorescence intensity is higher in stationary phase than in exponential growth phase. Merge of phase contrast and RFP channel in 13 x 13 µm fields of view are shown. Scale bars represent 1 µm. Representative images are shown (*n* = 3 biological replicates). Source data are provided as Source Data file.
m-n Assessment of interference capability of parental strain and *mtvS1 nonsense* **(m)**, *mtvS2 nonsense* and *mtvS1-2 nonsense* **(n)** mutants in a conjugation-based plasmid inhibition assay. Strains have a native type V-A CRISPR array or have the CRISPR array replaced by a spacer matching a protospacer on the conjugated plasmid (targeting spacer) or one that does not match (non-targeting spacer). Grey circles: parental strain. Turquoise circles: *mtvS1 nonsense* mutant. Purple circles: *mtvS2 nonsense* mutant. Blue circles: *mtvS1-2 nonsense* mutant. b.d.l.: below detection limit. Means with standard deviations are displayed (*n* = 3 biological replicates). Source data are provided as Source Data file.

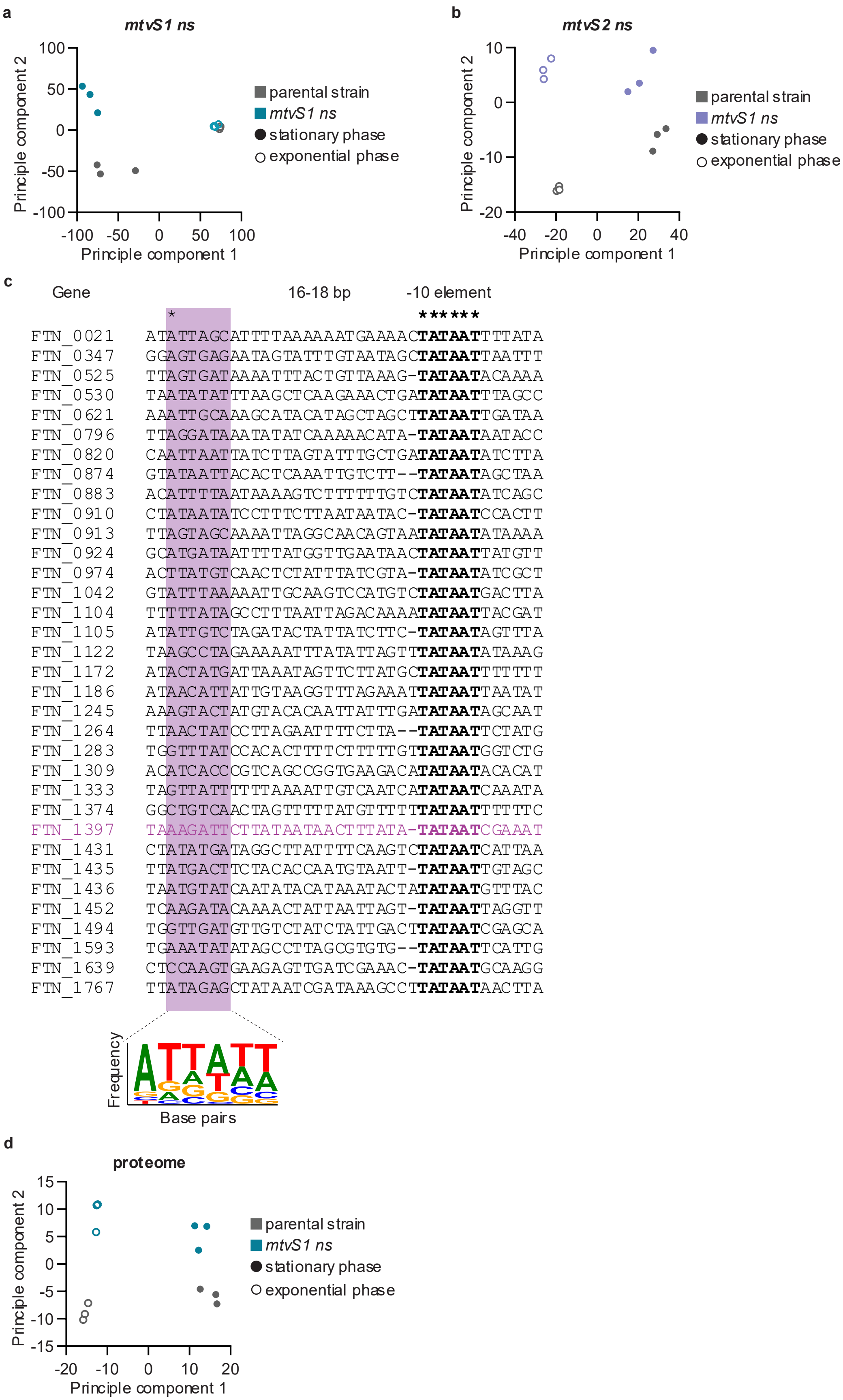
a
mtvS1 ns
Principle component 2
Principle component 1
parental strain
mtvS1 ns
stationary phase
exponential phase
b
mtvS2 ns
Principle component 2
Principle component 1
parental strain
mtvS1 ns
stationary phase
exponential phase
c
Gene
16-18 bp
-10 element
FTN_0021 ATATTAGCATTTTAAAAAATGAAAACTATAATTTTATA
FTN_0347 GGAGTGAGAATAGTATTTGTAATAGCTATAATTAATTT
FTN_0525 TTAGTGATAAAATTTACTGTTAAAG-TATAATACAAAA
FTN_0530 TAATATATTTAAGCTCAAGAAACTGATATAATTTAGCC
FTN_0621 AAATTGCAAAGCATACATAGCTAGCTTATAATTGATAA
FTN_0796 TTAGGATAAATATATCAAAAACATA-TATAATAATACC
FTN_0820 CAATTAATTATCTTAGTATTTGCTGATATAATATCTTA
FTN_0874 GTATAATTACACTCAAATTGTCTT--TATAATAGCTAA
FTN_0883 ACATTTTAATAAAAGTCTTTTTTGTCTATAATATCAGC
FTN_0910 CTATAATATCCTTTCTTAATAATAC-TATAATCCACTT
FTN_0913 TTAGTAGCAAAATTAGGCAACAGTAATATAATATAAAA
FTN_0924 GCATGATAATTTTATGGTTGAATAACTATAATTATGTT
FTN_0974 ACTTATGTCAACTCTATTTATCGTA-TATAATATCGCT
FTN_1042 GTATTTAAAAATTGCAAGTCCATGTCTATAATGACTTA
FTN_1104 TTTTTATAGCCTTTAATTAGACAAAATATAATTACGAT
FTN_1105 ATATTGTCTAGATACTATTATCTTC-TATAATAGTTTA
FTN_1122 TAAGCCTAGAAAAATTTATATTAGTTTATAATATAAAG
FTN_1172 ATACTATGATTAAATAGTTCTTATGCTATAATTTTTTT
FTN_1186 ATAACATTATTGTAAGGTTTAGAAATTATAATTAATAT
FTN_1245 AAAGTACTATGTACACAATTATTTGATATAATAGCAAT
FTN_1264 TTAACTATCCTTAGAATTTTCTTA--TATAATTCTATG
FTN_1283 TGGTTTATCCACACTTTTCTTTTTGTTATAATGGTCTG
FTN_1309 ACATCACCCGTCAGCCGGTGAAGACATATAATACACAT
FTN_1333 TAGTTATTTTTTAAAATTGTCAATCATATAATCAAATA
FTN_1374 GGCTGTCAACTAGTTTTTATGTTTTTTATAATTTTTTC
FTN_1397 TAAAGATTCTTATAATAACTTTATA-TATAATCGAAAT
FTN_1431 CTATATGATAGGCTTATTTTCAAGTCTATAATCATTAA
FTN_1435 TTATGACTTCTACACCAATGTAATT-TATAATTGTAGC
FTN_1436 TAATGTATCAATATACATAAATACTATATAATGTTTAC
FTN_1452 TCAAGATACAAAACTATTAATTAGT-TATAATTAGGTT
FTN_1494 TGGTTGATGTTGTCTATCTATTGACTTATAATCGAGCA
FTN_1593 TGAAATATATAGCCTTAGCGTGTG--TATAATTCATTG
FTN_1639 CTCCAAGTGAAGAGTTGATCGAAAC-TATAATGCAAGG
FTN_1767 TTATAGAGCTATAATCGATAAAGCCTTATAATAACTTA
Frequency
Base pairs
d
proteome
Principle component 2
Principle component 1
parental strain
mtvS1 ns
stationary phase
exponential phase

Supplementary Figure 3

Analysis of *F. novicida* U112 transcriptomic and proteomics data.

a-b Principal component analysis of parental strain and *mtvs1 nonsense* **(a)** and *mtvS2 nonsense* **(b)** mutants. Grey: parental strain. Turquoise: *mtvs1 nonsense* mutant. Purple: *mtvS2 nonsense* mutant. Filled circles: stationary phase samples. Empty circles: exponential phase samples. *n* = 3 biological replicates. Source data are provided as Source Data file.

c Promoter sequences of significantly downregulated operons for both the *mtvs1 nonsense* and *mtvS2 nonsense* mutants in stationary phase with TATAAT motifs were aligned to TATAAT motif (in bold). First genes of operons indicated. Bold stars: 100 % conserved sites. Star: 79 % conserved site. Magenta: promoter sequence of type V-A CRISPR-*cas* system. Web logo plot: frequency of base pairs 16-18 bp upstream of TATAAT motif.

d Principal component analysis of parental strain and *mtvs1 nonsense* mutant. Grey: parental strain. Turquoise: *mtvs1 nonsense*. Filled circles: stationary phase samples. Empty circles: exponential phase samples. *n* = 3 biological replicates. Source data are provided as Source Data file.

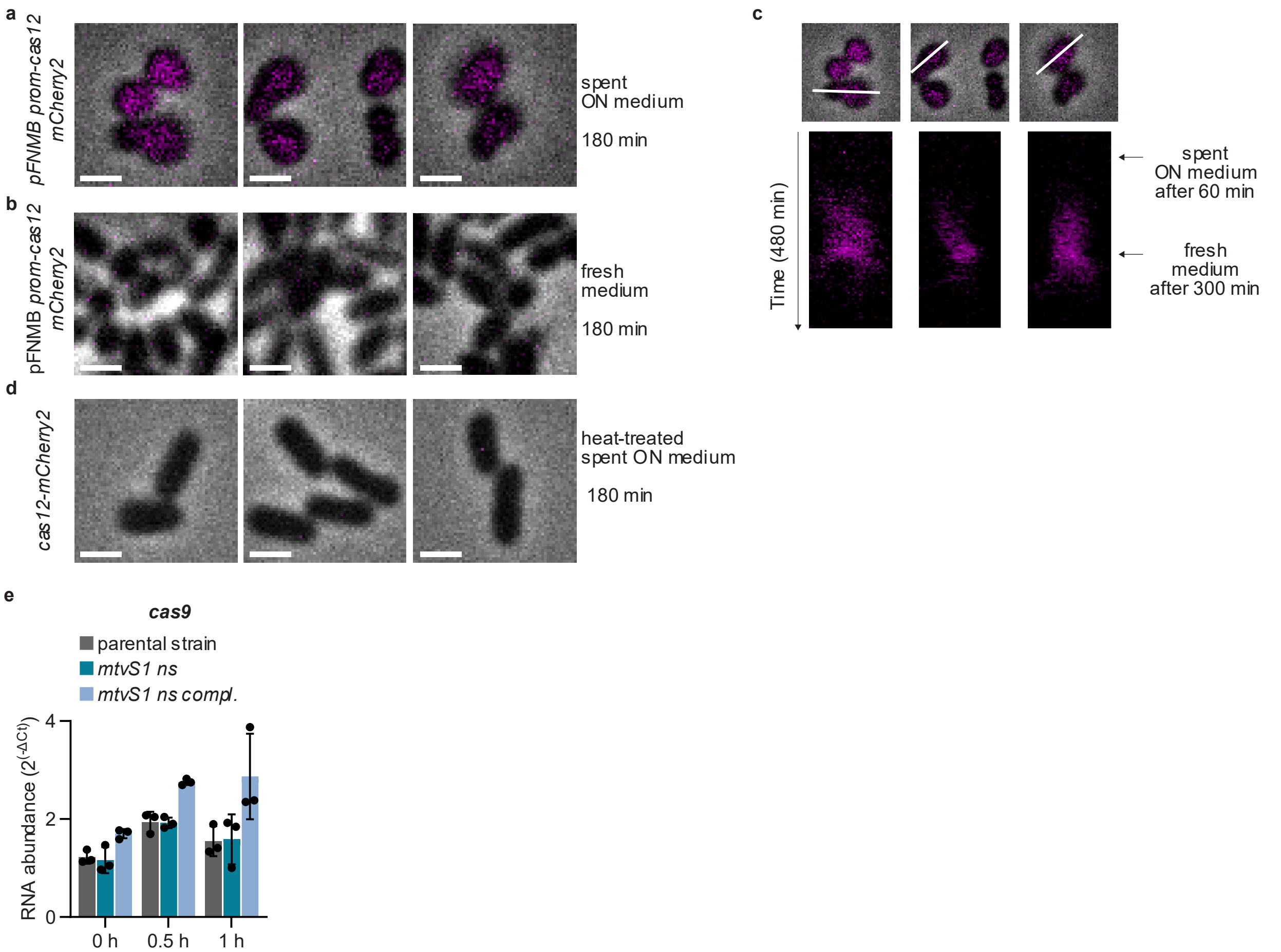

Supplementary Figure 4

Spent overnight medium is heat sensitive and does not affect *cas9* expression.

a-b Fluorescence microscopy of mCherry2 from *cas12* promoter on plasmid in a microfluidic plate. BHI was flown through for 1 h before switching to spent overnight medium of parental strain for 4h. Afterwards fresh BHI was flown through for 3 h. **(a)** Three examples of 240 min time point (180 min after switching to spent ON medium) and **(b)** three examples of 480 min (180 min after switching back to fresh BHI) are shown. Merge of phase contrast and RFP channel in 4 x 4 µm fields of view are shown. Same contrast settings as in **c** and **Supplementary Movie 6**. Scale bars represent 1 µ m. Representative images are shown (*n* = 2 biological replicates). Source data are provided as Source Data file.

c Examples of **a** and **b** were used to create time-resolved kymographs of RFP channel. Merge of phase contrast and RFP channel is shown (upper images). White lines show axis along which the kymographs of RFP channel were made (lower images). Same contrast settings as in **a-b** and **Supplementary Movie 6**. Representative images are shown (*n* = 2 biological replicates). Source data are provided as Source Data file.

d Fluorescence microscopy of Cas12-mCherry2 in the parental strain in a microfluidic plate. BHI was flown through for 1 h before switching to heat-treated spent overnight medium of parental strain for 4h. Afterwards fresh BHI was flown through for 3 h. Three examples of 240 min time

point (180 min after switching to spent ON medium) are shown. Merge of phase contrast and RFP channel in 4 x 4 μm fields of view are shown. Same contrast settings as in **Fig. 4c-g** and **Supplementary Movies 5, 7-8**. Scale bars represent 1 μm. Representative images are shown (*n* = 2 biological replicates). Source data are provided as Source Data file.

e Determination of mRNA levels for *cas9* after switching to spent overnight medium by RT-qPCR. Samples were taken 0 h, 0.5 h and 1 h after medium change. Grey bars: parental strain. Turquoise bars: *mtvS1 nonsense* mutant. Light blue bars: *mtvS1 nonsense* mutant with wild-type *mtvS1* at Tn7 insertion site. Means with standard deviations are displayed (*n* = 3 biological replicates). Significant differences were determined with a two-way ANOVA and Tukey's multiple comparison test (95 % confidence interval). Source data are provided as Source Data file.

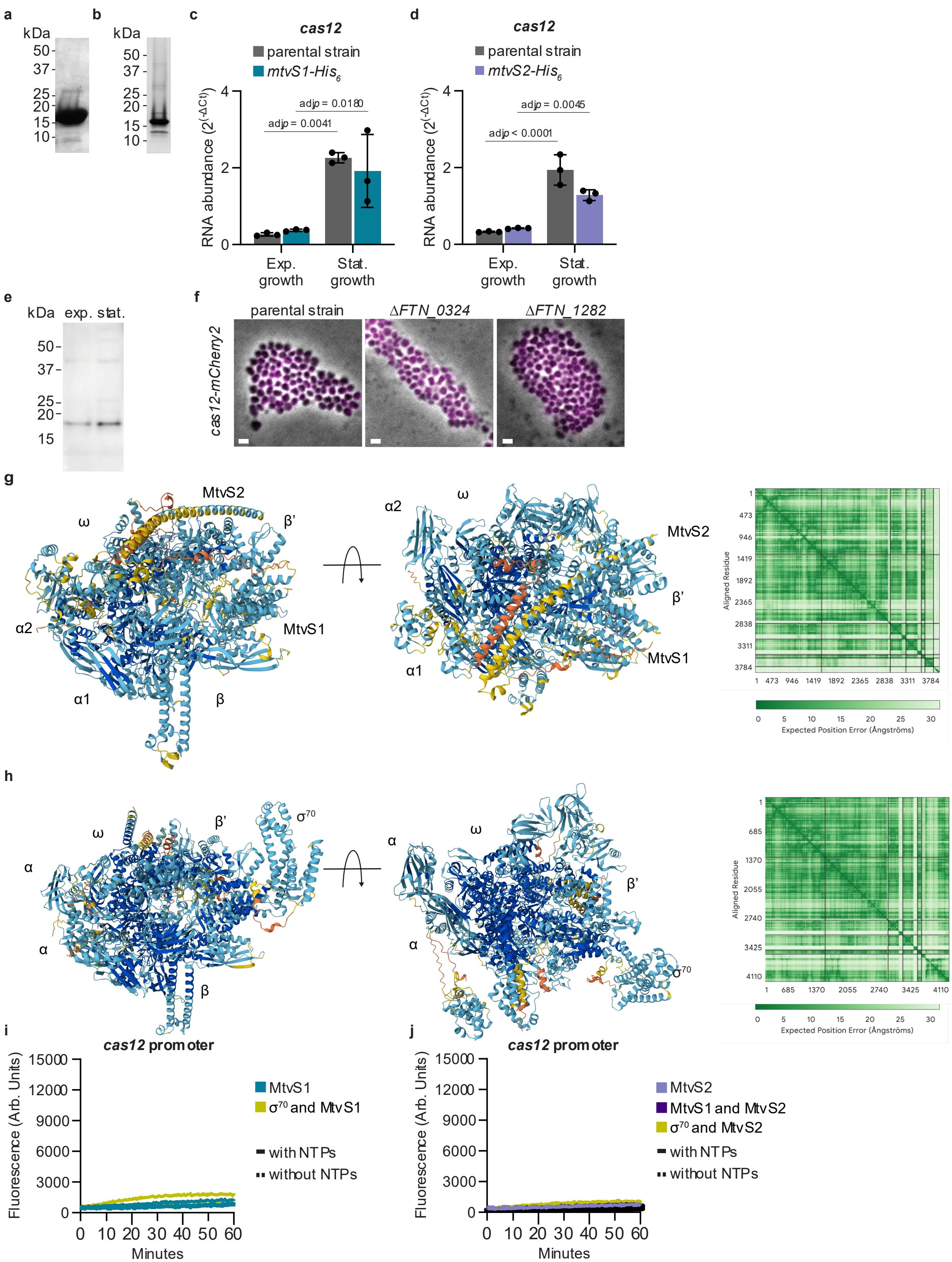

a
kDa
50
37
25
20
15
10
b
kDa
50
37
25
20
15
10
c
cas12
parental strain
mtvS1-His6
adjp = 0.0180
adjp = 0.0041
RNA abundance (2^(-ΔCt))
Exp. growth
Stat. growth
d
cas12
parental strain
mtvS2-His6
adjp = 0.0045
adjp < 0.0001
RNA abundance (2^(-ΔCt))
Exp. growth
Stat. growth
e
kDa exp. stat.
50
37
25
20
15
f
parental strain
ΔFTN_0324
ΔFTN_1282
cas12-mCherry2
g
MtvS2
ω
β'
α2
MtvS1
α1
β
α2
ω
MtvS2
β'
α1
MtvS1
Aligned Residue
Expected Position Error (Ångströms)
h
ω
β'
σ70
α
α
β
α
ω
β'
α
σ70
Aligned Residue
Expected Position Error (Ångströms)
i
cas12 promoter
Fluorescence (Arb. Units)
Minutes
MtvS1
σ70 and MtvS1
with NTPs
without NTPs
j
cas12 promoter
Fluorescence (Arb. Units)
Minutes
MtvS2
MtvS1 and MtvS2
σ70 and MtvS2
with NTPs
without NTPs

Supplementary Figure 5

MtvS1-His$_6$ and MtvS2-His$_6$ purification and AlphaFold 3 predictions.

a SDS-PAGE gel of MtvS1-His$_6$ purified with size-exclusion chromatography from *E. coli*. Representative image is shown (*n* = 2.) Source data are provided as Source Data file.

b SDS-PAGE gel of MtvS2-His$_6$ after dialysis after purification with 8 M urea from *E. coli*. Representative image is shown (*n* = 2). Source data are provided as Source Data file.

c-d Determination of mRNA levels for *cas12* by RT-qPCR. Grey bars: parental strain. Turquoise bars: *mtvS1-His$_6$* strain. Purple bars: *mtvS2-His$_6$* strain. Means with standard deviations are displayed (*n* = 3 biological replicates). Significant differences were determined with a two-way ANOVA and Tukey's multiple comparison test (95 % confidence interval). Source data are provided as Source Data file.

e Western blot probing for chromosomal MtvS1-His$_6$ in exponential growth and stationary phase lysates. *n* = 1. Source data are provided as Source Data file.

f Fluorescence microscopy of Cas12-mCherry2 in parental strain, *ΔFTN_0324* and *ΔFTN_1282* mutant. Comparable fluorescent intensities in stationary growth were detected. Merge of phase contrast and RFP channel in 13 x 13 µm fields of view are shown. Scale bars represent 1 µm. Representative images are shown (*n* = 2 biological replicates). Source data are provided as Source Data file.

g-h AlphaFold 3 stats for predictions of MtvS1 (A0A6I4RTR9) and MtvS2 (A0Q819) with *F. novicida* U112 RNA polymerase (RpoA1: A0Q4K8, RpoA2: A0Q7R6, RpoB: A0Q867, RpoC: A0Q866 and RpoZ: A0Q5J3) **(g)** and of *E. coli* σ^{70} (RpoD, P00579) with *E. coli* RNA polymerase (RpoB: P0A8V2, RpoC: P0A8T7, RpoA: P0A7Z4 and RpoZ: P0A800) (**h**). Models with pLDDT score overlaid from two viewing angles. Color code: dark blue, very high confidence; blue, confident; yellow, low confidence; orange, very low confidence. The predicted aligned error (PAE) plot is shown on the right. Overall prediction scores: ipTM = 0.79 and pTM = 0.82 **(g)** and ipTM = 0.82 and pTM = 0.84 **(h)**.

i-j Fluorescence based in vitro transcription assay with *cas12* promoter aptamer and *E. coli* RNA polymerase core enzyme with MtvS1-His$_6$ (turquoise) and with both MtvS1-His$_6$ and σ^{70} (light green) **(i)** or with MtvS2-His$_6$ (purple, both MtvS1-His$_6$ and MtvS2-His$_6$ (dark purple) or with σ^{70} (light green) **(j)**.Representative assays (mean and standard deviation, *n* = 3 technical replicates, repeated twice) with (solid line) and without (dashed line) NTPs added are shown. Source data are provided as Source Data file.

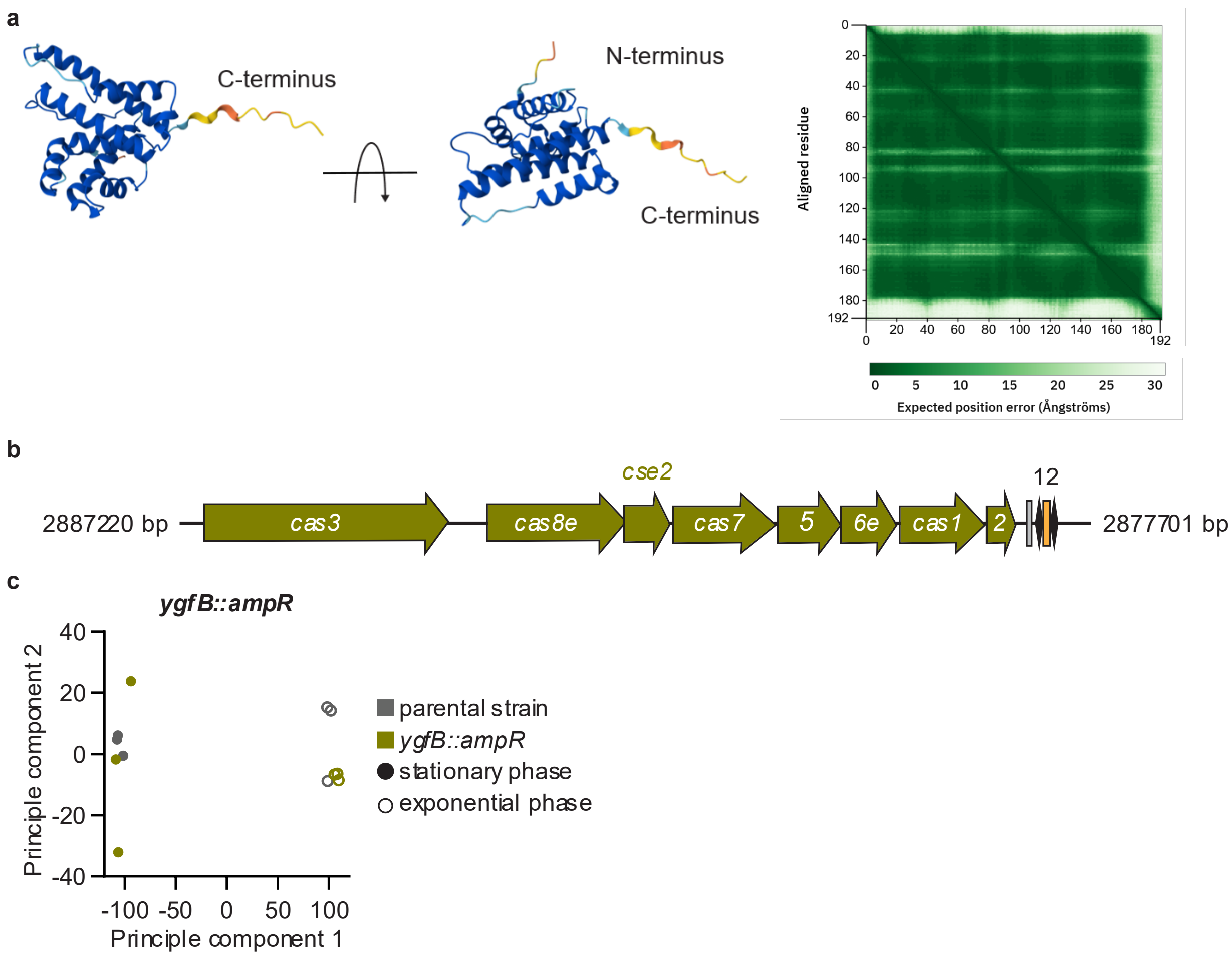

Supplementary Figure 6

Principal component analysis for *E. coli* transcriptomic data.

a AlphaFold 3 stats for predictions of *E. coli* YgfB (A8A450). Model with pLDDT score overlaid from two viewing angles. Color code: dark blue, very high confidence; blue, confident; yellow, low confidence; orange, very low confidence. The predicted aligned error (PAE) plot is shown on the right.

b Type I-E CRISPR- *cas* system in *E.coli* K-12 *substr*. BW25113. Olive green: *cas* and *cse* genes. Grey box: *leader/tracr sequence*. Black diamonds: *repeats*. Orange box: *spacers*. Drawn in scale.

c Principal component analysis of parental strain and *ygfB::ampR* mutant. Grey: parental strain. Olive green: *ygfB::ampR* mutant. Filled circles: stationary phase samples. Empty circles: exponential phase samples. *n* = 3 biological replicates. Source data are provided as Source Data file.

Supplementary Tables

Supplementary Table 1: Predicted interactions under 3 Å between RpoC and MtvS1.

RpoC (amino acid)	Type of interaction	MtvS1 (amino acid)
E40	side chain carboxyl hydrogen bond with backbone carbonyl	T52
T41	backbone carbonyl carbon interaction with lysine amine	K53
I42	backbone NH hydrogen bond with lysine amine	K53
N43	side chain amide nitrogen hydrogen bond with glutamic acid side chain carboxyl	E56
Y44	side chain phenol hydrogen bond with backbone carbonyl	E56
Y44	side chain phenol hydrogen bond with backbone carbonyl	E57
R45	side chain guanidinium hydrogen bond with carbonyl	E57
R268	side chain guanidinium hydrogen bond with side chain amide	Q19
R276	side chain guanidinium hydrogen bond with side chain carboxyl	E26
P286	proline ring carbon interaction with backbone carbonyl	L121
I288	backbone carbonyl interaction with side chain carbon	M139
E293	side chain carboxyl hydrogen bond to serine hydroxyl	S114
R295	side chain guanidinium hydrogen bond with backbone carbonyl	S142
R295	side chain guanidinium interactions with side chain phenol carbons	Y143
E299	side chain carboxyl hydrogen bond with side chain guanidinium	R110
R312	side chain carbons and backbone NH interactions with side chain carboxyl	E147
S317	serine hydroxyl hydrogen bond to lysine amine	K16

Supplementary Table 2: Strains used in this study.

Organism	Genotype	Plasmid	Relevant features	Source
Francisella novicida U112			Parental strain	Bei resources (NR-13)
	typeII_tet_spacer_targ		Type II-B wild-type CRISPR array was replaced with targeting spacer matching *tetR* on plasmid pFNMB2	This study
	typeII_tet_spacer_nontarg		Type II-B wild-type CRISPR array was replaced with non-targeting spacer matching *tetR* on plasmid pFNMB2	This study
	typeV_tet_spacer_targ		Type V-A wild-type CRISPR array was replaced with targeting spacer matching *tetR* on plasmid pFNMB2	This study
	typeV_tet_spacer_nontarg		Type V-A wild-type CRISPR array was replaced with non-targeting spacer matching *tetR* on plasmid pFNMB2	This study
	Δ*FTN_0710,* Δ*FTN_1487*		Deletion of *FTN_0710* and *FTN_1487*	This study
		pFNMB2 *mcs*	Empty vector control	This study
		pFNMB *prom-cas12 mCherry2*	mCherry2 expressed from *cas12* promoter	This study
	mtvS1 nonsense		Three stop codons introduced at position 11 of *mtvs1*	This study
	mtvS1 nonsense, *Tn7_mtvS1*		Wild-type *mtvS1* with endogenous promoter was introduced at the canonical Tn7 insertion site downstream of *glmS*	This study
	mtvS1 nonsense, *typeV_tet_spacer_targ*		Type V-A wild-type CRISPR array was replaced with targeting spacer matching *tetR* on plasmid pFNMB2	This study
	mtvs1 nonsense, *typeV_tet_spacer_nontarg*		Type V-A wild-type CRISPR array was replaced with non-targeting spacer matching *tetR* on plasmid pFNMB2	This study
	mtvS1-mCherry2		C-terminal chromosomal fusion of *mCherry2* to *mtvS1*	This study
	mtvS1-His$_6$		C-terminal chromosomal His$_6$-tag with a glycine linker	This study
	Δ*FTN_0392*, *typeV_tet_spacer_targ*		Type V-A wild-type CRISPR array was replaced with targeting spacer matching *tetR* on plasmid pFNMB2	This study
	Δ*FTN_0392*, *typeV_tet_spacer_nontarg*		Type V-A wild-type CRISPR array was replaced with non-targeting spacer matching *tetR* on plasmid pFNMB2	This study
	mtvS2 nonsense		Three stop codons introduced at position 11 of *mtvs2*	This study
	mtvS2 nonsense	pFNMB2 *mcs*	Empty vector control	This study
	mtvS2 nonsense	pFNMB *prom-upstream mtvS2*	Wild-type *mtvS2* with endogenous promoter upstream of gene on plasmid	This study
	mtvS1 nonsense, mtvS2 nonsense		Three stop codons introduced at position 11 of *mtvs1* and *mtvS2*	This study
	mtvS1 nonsense, mtvS2 nonsense, Δ*FTN_0710,* Δ*FTN_1487*		Three stop codons introduced at position 11 of *mtvs1* and *mtvS2* and deletion of *FTN_0710* and *FTN_1487*	This study
	mtvS2-His$_6$		C-terminal chromosomal His$_6$-tag with a glycine linker	This study
	cas9-msfGFP, *cas12-mCherry2*		C-terminal chromosomal fusion of *msfgfp* to *cas9* and c-terminal chromosomal fusion of *mCherry2* to *cas12*	This study
	cas9-msfGFP, *cas12-mCherry2*, *typeV_tet_spacer_targ*		Type V-A wild-type CRISPR array replaced with targeting spacer matching *tetR* on plasmid pFNMB2	This study
	cas9-msfGFP, *cas12-mCherry2*, *typeV_tet_spacer_nontarg*		Type V-A wild-type CRISPR array replaced with non-targeting spacer matching *tetR* on plasmid pFNMB2	This study
	cas9-msfGFP, *cas12-mCherry2*, *typeII_tet_spacer_targ*		Type II-B wild-type CRISPR array replaced with targeting spacer matching *tetR* on plasmid pFNMB2	This study
	cas9-msfGFP, *cas12-mCherry2*, *typeII_tet_spacer_nontarg*		Type II-B wild-type CRISPR array replaced with non-targeting spacer matching *tetR* on plasmid pFNMB2	This study

	cas9-msfGFP, *cas12-mCherry2*, *mtvs1 nonsense*		Three stop codons introduced at position 11 of *mtvS1*	This study
	cas9-msfGFP, *cas12-mCherry2*, Δ*FTN_0324*		Deletion of *FTN_0324*	This study
	cas9-msfGFP, *cas12-mCherry2*, Δ*FTN_0392*		Deletion of *FTN_0392*	This study
	cas9-msfGFP, *cas12-mCherry2*, Δ*FTN_1282*		Deletion of *FTN_1282*	This study
	cas12::mCherry2		*cas12* was replaced with *mCherry2*	This study
Escherichia coli K-12 *substr.* BW25113				Coli Genetic Stock Center at Yale University and [1]
		pAM38 (*red*)	λ Red recombineering plasmid	[2]
		pFNMB2 *mcs*	Empty vector control	This study
	ygfB::ampR		*ygfB* disrupted by ampicillin resistance cassette	This study
	ygfB::ampR	pFNMB2 *mcs*	Empty vector control	This study
	ygfB::ampR	pFNMB *prom mtvS1*	Wild-type *mtvS1* with endogenous promoter on plasmid	This study
	ygfB::ampR	pFNMB *prom ygfB*	Wild-type *ygfB* with endogenous promoter on plasmid	This study
Escherichia coli OneShotpir			Cloning strain	ThermoFisher
Escherichia coli JKE 201			Donor *E. coli* used for conjugation into *F. novicida*	[3]
		pFNMB2 *msfGFP*	Donor *E. coli* containing mobilizable *Francisella* plasmid pFNMB2 *msfGFP*	[4]
		pFNMB2 *msfGFP* *sp3_typeV_seedG3T_targ* *sp5_typeV_seedA3C_targ*	Plasmid containing protospacers 3 and 5 of native Type V CRISPR array carrying seed mutations G to T and A to C at position 3, respectively, and TTTG PAM	This study
Escherichia coli Rosetta (DE3)			Overexpression strain	Novagen
		pET21 *mtvS1-His$_6$*	Expression of MtvS1-His$_6$	This study
		pET21 *mtvS2-His$_6$*	Expression of MtvS2-His$_6$	This study
Escherichia coli BL21 (DE3)		pVS11/ p*E*crpo*ABC(-XH) Z)*, pACYC-Duet1_*EcoRpoZ*	Overexpression of *E. coli* RNA polymerase	[5,6]
		pSAD1403	Overexpression of *E. coli* σ^{70}	[7]

Supplementary Table 3: Plasmids, antibiotic resistance casettes and primers used to generate mutants.

Plasmid Name	Spacer/Peptide sequence	Primers	Sequence 5'-3' [base pairs]
pDMK3 *cas9-msfGFP*		FTN_0757-fusion_1.FOR	ATTGTCGACCCTCGAGCAAGCAGGTTTTGGATAAG
		FTN_0757-fusion_1.REV	GCGGCCGCATTATTAGATGTTTCATTATAAATACCT
		FTN_0757-fusion_2.FOR	TCTAATAATGCGGCCGCAGGA
		FTN_0757-msfGFP_2.REV	TACCAAGCATTTTGTAGAGCTCATCCATG
		FTN_0757-msfGFP_3.FOR	GCTCTACAAAATGCTTGGTATGAAATTAG
		FTN_0757-fusion_3.REV	ACAATTTGTGGAATTCCCGGGCGATACTCCTCCATTAGA
		FTN_0757-fusion_Det.FOR	TCTAAAAACCCAAAAATTACATGAG
		FTN_0757-fusion_Det.REV	TGTTCACCAGCAAATTCTCCAG
pDMK3 *cas12-mCherry2*		FTN_1397-fusion_1.FOR	CTGAATTGTCGACCCTCGAGCAAAAATATTAACAAAAACTATATTCTTT
		FTN_1397-mCherry2_1.REV	TGTACAAGTTATTTGAGTTCGTGCAGA
		FTN_1397-mCherry2_2.FOR	ACGAACTCAAATAACTTGTACAGCTCGT
		FTN_1397-fusion_2.REV	GAATAACGCGGCCGCAGGA
		FTN_1397-fusion_3.FOR	CGGCCGCGTTATTCCTATTCTGCACG
		FTN_1397-fusion_3.REV	CAATTTGTGGAATTCCCGGGGTGCTTAGAGCTTATCAG
		FTN_1397-fusion_Det.FOR	TCTCTTGCTGCTCTGGAGTT
		FTN_1397-fusion_Det.REV	AAAAGAGGGCGTTTCAAGGT
pDMK3 *cas12::mCherry2*		FTN_1397::mCherry2_1.FOR	CTTCTGAATTGTCGACCCTCGAGAGTCCGTTTTATCACATAAG
		FTN_1397::mCherry2_1.REV	TGTACAAGTAAGGTAGGATCAAAAATAATCA
		FTN_1397::mCherry2_2.FOR	TGATCCTACCTTACTTGTACAGCTCGTC
		FTN_1397::mCherry2_2.REV	AGTCTTTATCATGGTGAGCAAGGGC
		FTN_1397::mCherry2_3.FOR	GCTCACCATGATAAAGACTCCTTATAAAATT
		FTN_1397::mCherry2_3.REV	TCAGTAGCGGCCGCATTTTGATAAAGGTAAAAGAGC
		FTN_1397::mCherry2_Det.FOR	TCTCTTGCTGCTCTGGAGTT
		FTN_1397::mCherry2_Det.REV	ACACTTGCTGATGTTGGTGA
pDMK3 *typeII_tet_spacer_targ*	TCGCGATGACTTAGTAAAGCACATC TAAAACTTT	TypeII_spacer_1.FOR	CTGAATTGTCGACCCTCGAGATAGTTGACTGGGTTATAG
		TypeII_spacer_tet_targ_1.REV	AAAGTTTTAGATGTGCTTTACTAAGTCATCGCGAGTTTCAGTTGCTGAATT ATTTGGTAAACTACTGTTAGAGCCAAAACAAACCTAGTGT
		TypeII_spacer_tet_targ_2.FOR	TCGCGATGACTTAGTAAAGCACATCTAAAACTTTCTAACAGTAGTTTACCA AATAATTCAGCAACTGAAACTTAATTAATTTTGTAAATTA
		TypeII_spacer_2.REV	ATCTATCTAGAAGGGCCCCGTTTCACTAAATTCTTA
		TypeII_spacer_Det.FOR	ATGGGTTGCTCAAGGTGAAG
		TypeII_spacer_Det.REV	TCTCATGCTGGTTACGCTTG
pDMK3 *typeII_tet_spacer_nontarg*	AAAGTTTTAGATGTGCTTTACTAAG TCATCGCGA	TypeII_spacer_1.FOR	CTGAATTGTCGACCCTCGAGATAGTTGACTGGGTTATAG
		TypeII_spacer_tet_nontarg_1.REV	TCGCGATGACTTAGTAAAGCACATCTAAAACTTTGTTTCAGTTGCTGAATT ATTTGGTAAACTACTGTTAGAGCCAAAACAAACCTAGTGT
		TypeII_spacer_tet_nontarg_2.FOR	AAAGTTTTAGATGTGCTTTACTAAGTCATCGCGACTAACAGTAGTTTACCA AATAATTCAGCAACTGAAACTTAATTAATTTTGTAAATTA
		TypeII_spacer_2.REV	ATCTATCTAGAAGGGCCCCGTTTCACTAAATTCTTA
		TypeII_spacer_Det.FOR	ATGGGTTGCTCAAGGTGAAG
		TypeII_spacer_Det.REV	TCTCATGCTGGTTACGCTTG

pDMK3 *typeV_tet_spacer_targ*	TCTAACATCTCAATGGCTAAGGCGT CGAG	TypeV_spacer_1.FOR	TCTATCGCCTTCTTGACGAGTTCTTCTGAATTGTCGACCCTCGAGATGCT GTTTCCAAGC
		TypeV_spacer_tet_targ_1.REV	CTCGACGCCTTAGCCATTGAGATGTTAGAGTCTAAGAACTTTAAATAATTT GTCTGTATATTATTGATTTCTAAATTAGAATTTT
		TypeV_spacer_tet_targ_2.FOR	TCTAACATCTCAATGGCTAAGGCGTCGAGATCTACAACAGTAGAAATTAT TTAAAGTTCTTAGACCCGTTTTTGCCTAAATCAGC
		TypeV_spacer_2.REV	TTGTGAGCGGATAACAATTTGTGGAATTCCCGGGAGAGCTCCCTAGTTTG TGACTTTGTT
		TypeV_spacer_Det.FOR	GCCAGCGAATAAAAGTCCTG
		TypeV_spacer_Det.REV	CAAGCCTAGCGGAGCTAATG
pDMK3 *typeV_tet_spacer_nontarg*	CTCGACGCCTTAGCCATTGAGATG TTAGA	TypeV_spacer_1.FOR	TCTATCGCCTTCTTGACGAGTTCTTCTGAATTGTCGACCCTCGAGATGCT GTTTCCAAGC
		TypeV_spacer_tet_nontarg_1.REV	TCTAACATCTCAATGGCTAAGGCGTCGAGGTCTAAGAACTTTAAATAATTT GTCTGTATATTATTGATTTCTAAATTAGAATTTT
		TypeV_spacer_tet_nontarg_2.FOR	CTCGACGCCTTAGCCATTGAGATGTTAGAATCTACAACAGTAGAAATTATT TAAAGTTCTTAGACCCGTTTTTGCCTAAATCAGC
		TypeV_spacer_2.REV	TTGTGAGCGGATAACAATTTGTGGAATTCCCGGGAGAGCTCCCTAGTTTG TGACTTTGTT
		TypeV_spacer_Det.FOR	GCCAGCGAATAAAAGTCCTG
		TypeV_spacer_Det.REV	CAAGCCTAGCGGAGCTAATG
pDMK3 *mtvS1 nonsense*	MKNEKPNFED***ALK	FTN_1238_ns_1.FOR	CTTCTGAATTGTCGACCCTCGAGCCAAATAAGCCTACTCCT
		FTN_1238_ ns_1.REV	AGATTAGTAGTAGGCATTAAAAGTAATGCAA
		FTN_1238_ns_2.FOR	TACTTTTAATGCCTACTACTAATCTTCAAAATTAGGTTTTT
		FTN_1238_ns_2.REV	ATAACAATTTGTGGAATTCCCGGGAGAGCTCAGGTTACGCGGCCGCCCA CCCATGCCCATT
		FTN_1238_ns_Det.FOR	CCAACTATCCCACCCCCAAC
		FTN_1238_ns_Det.REV	TTTGCTGCTCATCAAAGGCT
		FTN_1238_ns_Det2.FOR	CCTACTACTAATCTTCAAAATTAG
pDMK3 *Tn7_mtvS1*		FTN_0485_Tn7_1.FOR	CTTCTGAATTGTCGACCCTCGAGAATGTGCCAAATAGTTCG
		FTN_1238_Tn7_1.REV	TTTAGGTATTAACTTGGAAAATAAATGATAAGAAAGTAAAA
		FTN_1238_Tn7_2.FOR	TTTATTTTCCAAGTTAATACCTAAATTTTTTCACTAT
		FTN_1238_Tn7_2.REV	ATTTAGCTAAGTTTAAACTCTTGATTACATAGCC
		FTN_1238_Tn7_3.FOR	ATCAAGAGTTTAAACTTAGCTAAATCTGTAACC
		FTN_0485_Tn7_3.REV	AGAGCTCAGGTTACGCGGCCGCGTATCAAGCAAGAGCTTAG
		FTN_0485_Tn7_Det.FOR	TCGTTGCATGTGGAACTAGCT
		FTN_0485_Tn7_Det.REV	TGGTGAAACACTAGCTGAAGGT
pDMK3 *mtvS1-mCherry2*		FTN_1238-tag_1.FOR	TCTATCGCCTTCTTGACGAGTTCTTCTGAATTGTCGACCCTCGAGTAGGT AACATCGCTAGTACAC
		FTN_1238-mCherry2_1.REV	CTGTACAAGTTATAGTTATCAGGAGTAGGCTTATT
		FTN_1238-mCherry2_2.FOR	ACTCCTGATAACTATAACTTGTACAGCTCG
		FTN_1238-mCherry2_2.REV	AAAAAATTGCGGCCGCAGGA
		FTN_1238-mCherry2_3.FOR	CGGCCGCAATTTTTTCACTATCAATAAGTAAAAC
		FTN_1238-mCherry2_3.REV	AGAGCTCAGGTTACGCGGCCGCGATTACATAGCCATTATAAATCAC
		FTN_1238-tag_Det.FOR	GCCGAAGCGATCTTGGACTA
		FTN_1238_ns_Det.REV	TTTGCTGCTCATCAAAGGCT

pDMK3 *mtvS1-His*$_6$	Added: GGTCACCACCACCACCACCAC	FTN_1238-tag_1.FOR	TCTATCGCCTTCTTGACGAGTTCTTCTGAATTGTCGACCCTCGAGTAGGTAACATCGCTAGTACAC
		FTN_1238-His_1.REV	GGTCACCACCACCACCACCACTAGGTATTAACATGAATACTAAATATGATGTG
		FTN_1238-His_2.FOR	CTAGTGGTGGTGGTGGTGGTGACCAATTTTTTCACTATCAATAAGTAAAAC
		FTN_1238-His_2.REV	ATAACAATTTGTGGAATTCCCGGGAGAGCTCAGGTTACGCGGCCGCATGGGCGATGTATATTTCTTTTATGATGTGAAT
		FTN_1238-tag_Det.FOR	GCCGAAGCGATCTTGGACTA
		FTN_1238_ns_Det.REV	TTTGCTGCTCATCAAAGGCT
		His_Det.REV	CACCACCACCACCACCAC
pDMK3 *mtvS2 nonsense*	MKKPNFDINK***PND	FTN_1519_ns_1.FOR	CTTCTGAATTGTCGACCCTCGAGCAGACATTTTTCGC
		FTN_1519_ns_1.REV	ATTAATAAATAATAATAACCTAATGATGATTTAGTAGGC
		FTN_1519_ns_2.FOR	ATTAGGTTATTATTATTTATTAATATCAAAATTAGGTTTCTTCA
		FTN_1519_ns_2.REV	AGAGCTCAGGTTACGCGGCCGCGCACCACAAATTCG
		dFTN_1519_Det.FOR	CCCTAGCAATAACTCGTTGGGT
		dFTN_1519_Det.REV	AGAGAATGCAAGCTTAGCAAAGT
pDMK3 *mtvS2-His*$_6$	Added: GGTCACCACCACCACCACCAC	FTN_1519_ns_1.FOR	CTTCTGAATTGTCGACCCTCGAGCAGACATTTTTCGC
		FTN_1519-His_1.REV	AACGGTCACCACCACCACCACCACTAATAAATGCAAAGTCAGTCG
		FTN_1519-His_2.For	ATTAGTGGTGGTGGTGGTGGTGACCGTTTTCTCTAATTTTAGCTAACTC
		FTN_1519_ns_2.REV	ATAACAATTTGTGGAATTCCCGGGAGAGCTCAGGTTACGCGGCCGCATGGGCGATGTATATTTCTTTTATGATGTGAAT
pDMK3 Δ*FTN_0324*	MITVTNDKWTIETELTLKTVLSLAKVHGAKTILEEYIN*	dFTN_0324_1.FOR	CTTCTGAATTGTCGACCCTCGAGAATATTAGTACTGTTAACAAAGC
		dFTN_0324_1.REV	CTAAAAACTGTTCTTTCTTTAGCAAAGGTG
		dFTN_0324_2.FOR	TGCTAAAGAAAGAACAGTTTTTAGCGTTAATT
		dFTN_0324_2.REV	AGAGCTCAGGTTACGCGGCCGCCATAGATTAACTTTAAGAGCTTT
		dFTN_0324_Det.FOR	AGTGTTGGTACGACAGGTGC
		dFTN_0324_Det.REV	ACACTTTTGTTGCAGGCAAAGA
pDMK3 Δ*FTN_0392*	MRITLKQLQVFIEKDINGCDIRLENFSI*	dFTN_0392_1.FOR	TCTATCGCCTTCTTGACGAGTTCTTCTGAATTGTCGACCCTCGAGAAGTGTTCTTACGTACA
		dFTN_0392_1.REV	CTCTAAAACAGCTTCAAGTGTTTATAGAGAAAGATATAAATGGCTG
		dFTN_0392_2.FOR	TTTATATCTTTCTCTATAAACACTTGAAGCTGTTT
		dFTN_0392_2.REV	ATAACAATTTGTGGAATTCCCGGGAGAGCTCAGGTTACGCGGCCGCGTGAGACGAACCAAAAAC
		dFTN_0392_Det.FOR	TGCAAATTCACTATAGCGCCA
		dFTN_0392_Det.REV	TTGCAACATCCTCAACCCCA
pDMK3 Δ*FTN_0710*	MKKNNSQFSTQKAYELGVNNYD*	dFTN_0710_1.FOR	TCAGTACTCGAGTTATACATTATAAAGAAGTTTTTATCCC
		dFTN_0710_1.REV	GTAAGCTTTTTGAGTTGAAAATTGAGAATTGTT
		dFTN_0710_2.FOR	AATTTTCAACTCAAAAAGCTTACGAGCT
		dFTN_0710_2.REV	TCAGTAGCGGCCGCGCTTGTAATGAAAATTCATGC
		dFTN_0710_Det.FOR	AAGAAGCTATGCTCTCCTTTTGA
		dFTN_0710_Det.REV	AAGCTATCGAAGGTGGCAAA

pDMK3 Δ*FTN_1282*	MGHLERLKIFIKAAHLSAYIEKNLPKIEL*	dFTN_1282_1.FOR<br>dFTN_1282_1.REV<br>dFTN_1282_2.FOR<br>dFTN_1282_2.REV<br>dFTN_1282_Det.FOR<br>dFTN_1282_Det.REV	CTTCTGAATTGTCGACCCTCGAGGTTTTAGCTGTGTTTAAACC<br>GCGCTTAGATGTGCTGCTTTTATAAATATC<br>CAGCACATCTAAGCGCTTATATAGAGAAA<br>AGAGCTCAGGTTACGCGGCCGCGTTGTGATGGTACAGG<br>ATCCTGCAGCAACAGAACCC<br>AGAGCAGCTCGAGGTAGTGA
pDMK3 Δ*FTN_1487*	MLFEKQQYQEDCVNN ELSNILQECQK*	dFTN_1487_1.FOR<br>dFTN_1487_1.REV<br>dFTN_1487_2.FOR<br>dFTN_1487_2.REV<br>dFTN_1487_Det.FOR<br>dFTN_1487_Det.REV	TCAGTACTCGAGTCCTTGGAATATAGTTAAGTAATTAATT<br>AGATTGTGTAAATAATGAATTATCTAATATTTTACAGGAGTG<br>AAATATTAGATAATTCATTATTTACACAATCTTCTTGATAT<br>TCAGTAGCGGCCGCCGTTAGATATAGTTTTAGCAG<br>CTGATGCAGCTTGGGAAAAT<br>TGAAACAGTATGGGCTAGTCAGA
pFNMB2 *mcs*	Multiple cloning site	pFNMB2_mcs.FOR<br>pFNMB2_mcs.REV<br>pFNMB_Det.FOR<br>pFNMB_Det.REV	CTGCAGATTTAAGAAGGAGATACGCGTACTAGTGGGGCC<br>CGGGTTATTTGTAGAGCTCAGGTTACGCGGC<br>TCATAGAAGCTTGCATGCCTG<br>AGACCCCACACTACCATCG
pFNMB *prom-cas12 mCherry2*		pFNMB_prom-FTN_1397_1.FOR<br>FTN_1397_Prom_mCherry2_1.REV<br>FTN_1397_Prom_mCherry2_2.FOR<br>pFNMB_mCherry2.REV<br>pFNMB_Det.FOR<br>pFNMB_Det.REV	TTAATCAGATAAAATATTTCTAGAACTAGTCTAGCTAGTTTAAAAGGACAA<br>TGCTCACCATGATAAAGACTCCTTATAAAATT<br>GTCTTTATCATGGTGAGCAAGGGC<br>TGTTTTATCAGACCGCCCCGGGTTACTTGTACAGCTCGTCCA<br>TCATAGAAGCTTGCATGCCTG<br>AGACCCCACACTACCATCG
pFNMB *prom-upstream mtvS2*		pFNMB_upstream-Prom-FTN_1519.FOR<br>pFNMB_FTN_1519.REV<br>pFNMB_Det.FOR<br>pFNMB_Det.REV	TTAATCAGATAAAATATTTCTAGAACTAGTTATATGGAAACAATGGCTTC<br>TGTTTTATCAGACCGCCCCGGGTTAGTTTTCTCTAATTTTAGCTAACT<br>TCATAGAAGCTTGCATGCCTG<br>AGACCCCACACTACCATCG
pFNMB *prom mtvS1*		pFNMB_Prom-FTN_1238.FOR<br>pFNMB_FTN_1238.REV<br>pFNMB_Det.FOR<br>pFNMB_Det.REV	TTAATCAGATAAAATATTTCTAGAACTAGTAAACTCTTGATTACATAGCCATTA<br>TGTTTTATCAGACCGCCCCGGGCTAAATTTTTTCACTATCAATAAGT<br>TCATAGAAGCTTGCATGCCTG<br>AGACCCCACACTACCATCG
pFNMB *prom ygfB*		pFNMB_Prom-b2909.FOR<br>pFNMB_b2909.REV<br>pFNMB_Det.FOR<br>pFNMB_Det.REV	TTAATCAGATAAAATATTTCTAGAACTAGTTGATGAGAAGAGACAAGC<br>TGTTTTATCAGACCGCCCCGGGTTAGTGTAGAGTCGGTTTTT<br>TCATAGAAGCTTGCATGCCTG<br>AGACCCCACACTACCATCG
pFNMB2 *msfGFP sp3_typeV_seedG3T_targ sp5_typeV_seedA3C_targ*	Protopacer 3 with seed mutation G3C and TTTG PAM: TTTGGTTTAGAGCCTTTTGTATTAGTAGCCG<br>Protopacer 5 with seed mutation A3C and TTTG PAM: TTTGAGCTTAAAAGGTAATTCTATCTTGTTGAG	pFNMB_TypeV_sp3_seedC3T.FOR<br>pFNMB_TypeV_sp3_seedC3T.REV<br>pFNMB_TypeV_sp5_seedA3C.FOR<br>pFNMB_TypeV_sp5_seedA3C.REV	CGGCTACTAATACAAAAGGCTCTAAACCAAATTCTAGAAATATTTTATCTGATTAA<br>GTTTAGAGCCTTTTGTATTAGTAGCCGCTAGTTTAAGACCCACTTTCACATT<br>CTCAACAAGATAGAATTACCTTTTAATCTCAAAGAGCTCATCCATGCCGTGCG<br>AGCTTAAAAGGTAATTCTATCTTGTTGAGCCCGGGGCGGTCTGA

pFNMB *promoterless aptamer*	See Supplementary Table 11	pFNMB_Promoterless-aptamer.FOR	TTAATCAGATAAAATATTTCTAGAACTAGTATGTCAATTTATCAAGAATTTGT
		pFNMB_aptamer.REV	TGTTTTATCAGACCGCCCCGGGGAGAGCGTTCACCG
		pFNMB_Det.FOR	TCATAGAAGCTTGCATGCCTG
		pFNMB_Det.REV	AGACCCCACACTACCATCG
pFNMB *cas9 promoter aptamer*	See Supplementary Table 11	pFNMB_Prom-cas9-aptamer.FOR	TTAATCAGATAAAATATTTCTAGAACTAGTTTAAATTTATAACCTATAAAGAAATTTCAG
		Prom-cas9-aptamer_1.REV	GATAAATTGACATAAAATGACCTCTTTT
		Prom-cas9-aptamer_2.FOR	AGAGGTCATTTTATGTCAATTTATCAAG
		pFNMB_aptamer.REV	TGTTTTATCAGACCGCCCCGGGGAGAGCGTTCACCG
		pFNMB_Det.FOR	TCATAGAAGCTTGCATGCCTG
		pFNMB_Det.REV	AGACCCCACACTACCATCG
pFNMB *cas12 promoter aptamer*	See Supplementary Table 11	pFNMB_Prom-cas12-aptamer.FOR	TTAATCAGATAAAATATTTCTAGAACTAGTAGATGAAACGCTAAAAATATATG
		Prom-cas12-aptamer_1.REV	ATAAATTGACATGATAAAGACTCCTTA
		Prom-cas12-aptamer_2.FOR	GGAGTCTTTATCATGTCAATTTATCAAG
		pFNMB_aptamer.REV	TGTTTTATCAGACCGCCCCGGGGAGAGCGTTCACCG
		pFNMB_Det.FOR	TCATAGAAGCTTGCATGCCTG
		pFNMB_Det.REV	AGACCCCACACTACCATCG
pET21 *mtvS1-His*$_6$		pET21.FOR	GGTCACCACCACCACCACCACTG
		pET21.REV	ATGTATATCTCCTTCTTAAAGTTAAACAAAATTATTTCTAGAGGG
		pET21_FTN_1238.FOR	TTAACTTTAAGAAGGAGATATACATATGGGCGATGTATATTTC
		pET21_FTN_1238-His.REV	GTGGTGGTGGTGACCAATTTTTTCACTATCAATAAGTAAAAC
pET21 *mtvS2-His*$_6$		pET21_stop.FOR	TAATAGGTAAGCTGAATCCGGCTGCTAACAAAGCC
		pET21.REV	ATGTATATCTCCTTCTTAAAGTTAAACAAAATTATTTCTAGAGGG
		pET21_FTN_1519-His.FOR	TTAACTTTAAGAAGGAGATATACATATGAAGAAACCTAATTTTGATATTAA
		pET21_FTN_1519-His_stop.REV	TCAGCTTACCTATTATCAGTGGTGGTGGTGGTGGTGACCGTTTTCTCTAATTTTAGCTAACTC
	Ampicillin resistance cassette with 50 bp homology arms to *ygfB*	ygfB::ampR.FOR	GCCATGAGCTGTCATCGTTACCGCCACATATCATCCCGCTGATTAAACCATTACCAATGCTTAATCAGTGAG
		ygfB::ampR.REV	AACCAGTATCTGAACCAACAAGGGACGGGTCTGACCCCAGCTGAGATGCACGCGGAACCCCTATTT
		ygfB::ampR_Det.FOR	GCATCAGCCCGATCGAAAAC
		ygfB::ampR_Det.REV	AAGCCCTTTTCTGGTCCACC

Supplementary Table 4: Sequencing and EDGE-Pro statistics for comparison of gene expression between exponentially growing and stationary phase cells of parental strain and *mtvS1 nonsense* mutant. RPKM reads were calculated as "Mapped reads"- "rRNA reads" and were the basis to calculate RPKM values.

	**Parental strain Exponential growth Replicate 1**	**Parental strain Exponential growth Replicate 2**	**Parental strain Exponential growth Replicate 3**	**Parental strain Stationary phase Replicate 1**	**Parental strain Stationary phase Replicate 2**	**Parental strain Stationary phase Replicate 3**
Total reads	2986954	2712522	2765874	6025619	4080967	4575747
Mapped reads	2913730	2612638	2669732	5818536	3806447	4336287
RPKM reads	2899145	2463275	2644707	5766824	2503016	3178846
rRNA reads	14585	149363	25025	51712	1303431	1157441
Unique reads	2784875	2363729	2528180	5747217	2500232	3176617
Multiple reads	128855	248909	141552	71319	1306215	1159670

	***mtvS1 nonsense* Exponential growth Replicate 1**	***mtvS1 nonsense* Exponential growth Replicate 2**	***mtvS1 nonsense* Exponential growth Replicate 3**	***mtvS1 nonsense* Stationary phase Replicate 1**	***mtvS1 nonsense* Stationary phase Replicate 2**	***mtvS1 nonsense* Stationary phase Replicate 3**
Total reads	2061676	2567719	2530234	5902835	5486068	6047529
Mapped reads	1967414	2482642	2466641	5669047	5222292	5744518
RPKM reads	1965250	2477719	2447727	5446356	3641674	3529729
rRNA reads	2164	4923	18914	222691	1580618	2214789
Unique reads	1892514	2383803	2334436	5438902	3636613	3525217
Multiple reads	74900	98839	132205	230145	1585679	2219301

Supplementary Table 5: Sequencing and EDGE-Pro statistics for comparison of gene expression between exponentially growing and stationary phase cells of parental strain and *mtvS2 nonsense* mutant. RPKM reads were calculated as "Mapped reads"- "rRNA reads" and were the basis to calculate RPKM values.

	**Parental strain Exponential growth Replicate 1**	**Parental strain Exponential growth Replicate 2**	**Parental strain Exponential growth Replicate 3**	**Parental strain Stationary phase Replicate 1**	**Parental strain Stationary phase Replicate 2**	**Parental strain Stationary phase Replicate 3**
Total reads	8343386	3839674	3456171	3661319	3203981	2860169
Mapped reads	7896369	3615019	3233160	3347984	2996038	2697736
RPKM reads	7881916	3604581	3226269	2284452	2416302	2611472
rRNA reads	14453	10438	6891	1063532	579736	86264
Unique reads	7654326	3511513	3125523	2263161	2404676	2595038
Multiple reads	242043	103506	107637	1084823	591362	102698

	***mtvS2 nonsense* Exponential growth Replicate 1**	***mtvS2 nonsense* Exponential growth Replicate 2**	***mtvS2 nonsense* Exponential growth Replicate 3**	***mtvS2 nonsense* Stationary phase Replicate 1**	***mtvS2 nonsense* Stationary phase Replicate 2**	***mtvS2 nonsense* Stationary phase Replicate 3**
Total reads	3808430	4384771	3230924	3195029	3369067	2376528
Mapped reads	3639036	4199206	3072765	3028047	3201878	2282368
RPKM reads	3630774	4124349	3067361	1969882	2733793	2107863
rRNA reads	8262	74857	5404	1058165	468085	174505
Unique reads	3565246	4056135	3013881	1961332	2719408	2096063
Multiple reads	73790	143071	58884	1066715	482470	186305

Supplementary Table 6: Sequencing and EDGE-Pro statistics for comparison of gene expression between exponentially growing and stationary phase cells of parental *E. coli* strain and *ygfB::ampR* mutant. RPKM reads were calculated as "Mapped reads"- "rRNA reads" and were the basis to calculate RPKM values.

	Parental strain Exponential growth Replicate 1	Parental strain Exponential growth Replicate 2	Parental strain Exponential growth Replicate 3	Parental strain Stationary phase Replicate 1	Parental strain Stationary phase Replicate 2	Parental strain Stationary phase Replicate 3
Total reads	36054722	34304644	36224450	32786560	33084758	33454216
Mapped reads	35315609	33583473	35426675	30923012	30522365	30775577
RPKM reads	34848479	33557224	34954563	30835863	30367552	30524275
rRNA reads	467130	26249	472112	87149	154813	251302
Unique reads	34320171	33035446	34390746	30814664	30329727	30489307
Multiple reads	995438	548027	1035929	108348	192638	286270

	ygfB::ampR Exponential growth Replicate 1	*ygfB::ampR* Exponential growth Replicate 2	*ygfB::ampR* Exponential growth Replicate 3	*ygfB::ampR* Stationary phase Replicate 1	*ygfB::ampR* Stationary phase Replicate 2	*ygfB::ampR* Stationary phase Replicate 3
Total reads	35581174	35065810	34079434	35337534	36245370	36009826
Mapped reads	34997515	32587796	33452416	33606379	34180348	33920664
RPKM reads	34981316	32571859	33440952	31792241	34120083	33853745
rRNA reads	16199	15937	11464	1814138	60265	66919
Unique reads	34390494	32034671	32857912	31899435	34092439	33830137
Multiple reads	607021	553125	594504	1706944	87909	90527

Supplementary Table 7: Primers used for RT-qPCR.

Primers	Sequence 5'-3' [base pairs]
FTN_0757_qPCR. FOR	ATGCTAACACTTGTGCAGTTTG
FTN_0757_qPCR.REV	GTCTCTGAGCTTTGGCACTTA
FTN_1397_qPCR.FOR	AAGCTCTGCCTCACCATTTAG
FTN_1397_qPCR.REV	AAGGGCGACCAAATCTACATAC
FTN_1396_qPCR.FOR	GCCCATCTCTTGGAGACAAA
FTN_1396_qPCR.REV	GGGCTTACTTGTGGAGAGAAA
FTN_1395_qPCR.FOR	TATTCTAGGTTTGCGCCCTTG
FTN_1395_qPCR.REV	CAAGCCTAGCGGAGCTAATG
FTN_1394_qPCR.FOR	TCACATCTTTATCTGGAGCATT
FTN_1394_qPCR.REV	TGGTGTACGTTTACAATATTCGG
FTN_1238_qPCR.FOR	CACCCTCTTCAATCGGCTTAG
FTN_1238_qPCR.REV	GGACTGCTTTGTGCTTTGTTT
FTN_1519_qPCR.FOR	ATTCTTCCTGCAAAGCACATATC
FTN_1519_qPCR.REV	AGAATGGATCTTGAGTGTGAAGT
FTN_1594_qPCR.FOR	GAGTCTATCTCTGCGACGAAAC
FTN_1594_qPCR.REV	GTAACAAGAGCTATGAAAGCCTTAAC
b2756_qPCR.REV	CAGGTTGGTGTTCCACTCTATT
b2756_qPCR.REV	CAGGTTGGTGTTCCACTCTATT
b2758_qPCR.FOR	CCTGAAGTTGAGCGAGGTTAAT
b2758_qPCR.REV	GGTTCACCGCTGTAGATGATT
b2760_qPCR.FOR	ACGTTGTTACAGGTGTTCCTC
b2760_qPCR.REV	GCACTGCGATTGCGTTATTC
b3699_qPCR.FOR	TGTTCCTGCTTGCCTTTCT
b3699_qPCR.REV	GCTGCTGTTGACCTTCTTCTA

Supplementary Table 8: Template switch oligo and primers used for 5' RACE.

Template switch oligo/ Primers	Sequence 5'-3' [base pairs]
Template switch oligo	GCTAATCATTGCAAGCAGTGGTATCAACGCAGAGTACATRGRGRG
FTN_1397-RT.REV	CTTTAGCTCTTTTCTCATCATCT
TSO.FOR	CATTGCAAGCAGTGGTATCAAC
FTN_1397-RT_PCR2.REV	ACCTCTTGCTTTTATGTTTTCAAGTGTTTTACCCT

Supplementary Table 9: Primers used for promoter sequence pull-down.

Primers	Modification	Sequence 5'-3' [base pairs]
Promoter-FTN_1397.FOR		CTAGCTAGTTTAAAAGGACAA
Promoter-FTN_1397_biotin.REV	5' Biotin-TEG	TAACTCAAATCTTAGAGTTTTACTT
FTN_1397_pull-down.FOR		GCTGTACCAATAACACTATAAT
FTN_1397_biotin.REV	5' Biotin-TEG	AGAAAAATCTATTAAAGAAACACTATC

Supplementary Table 10: Primers used for EMSA.

Sequences and Primers	Sequence 5'-3' [base pairs]
Promoter-FTN_1397.FOR	CTAGCTAGTTTAAAAGGACAA
Prom-FTN_1397_EMSA.REV	TAACTCAAATCTTAGAGTTTTACTT
FTN_1397_pull-down.FOR	GCTGTACCAATAACACTATAAT
FTN_1397_EMSA.REV	TGATGTAGTTACAACGATGC

Supplementary Table 11: Aptamer sequences used for *in vitro* transcription assays.

Aptamers and Primers	Sequence 5’-3’ [base pairs]
Promoterless aptamer	ATGTCAATTTATCAAGAATTTGTTAATAAATAGACGCGACCGAAATGGTGAAGGACGGGTCCAGTGCTTCGGCACTGTTGAGTAGAGTGTGAGCTCCGTA<br>ACTGGTCGCGTCTAGTTTAAGTAAAACTCTAAGATTTGAGTTAACCAGGCATCAAATAAAACGAAAGGCTCAGTCGAAAGACTGGGCCTTTCGTTTTATCT<br>GTTGTTTGTCGGTGAACGCTCTCC
Promoterless-aptamer.FOR	ATGTCAATTTATCAAGAATTTGTT
Aptamer.REV	GGAGAGCGTTCACCGACAAA
cas9 promoter aptamer	TTAAATTTATAACCTATAAAGAAATTTCAGTGAGTTATTTACTAAAATTCATAGAATCATTCATACTTTCTAGAAATCAAATAATATTTACATAAATCGTAGCTA<br>ATACTGACAATATTTCTTTCAAAATGATTTATAAAATTTGTAATTTTAGATTAGTTTTACTATTAGGAAAATTGATAGTTATATAGAGCTTTATGAAGTAGAATT<br>AATGTATAAGTTTAACTACTTTTCCATTTTTTAATTTGATTTTGATTTTAAATGCTGGTTTAATGCTTTATTAAACCAATGATAAACGTATGTGAAGCTAGTTC<br>CTAAAAGCTTTTATCCAAGTTTATAGATTTATGATTAAAAACTTATATCAAAATATTATTCTATACCGAGTAAAAGAGGTCATTTTATGTCAATTTATCAAGAA<br>TTTGTTAATAAATAGACGCGACCGAAATGGTGAAGGACGGGTCCAGTGCTTCGGCACTGTTGAGTAGAGTGTGAGCTCCGTAACTGGTCGCGTCTAGTT<br>TAAGTAAAACTCTAAGATTTGAGTTAACCAGGCATCAAATAAAACGAAAGGCTCAGTCGAAAGACTGGGCCTTTCGTTTTATCTGTTGTTTGTCGGTGAAC<br>GCTCTCC
FTN_0757-aptamer_1.FOR	TTAAATTTATAACCTATAAAGAAATTTCAG
FTN_0757-aptamer_1.REV	GATAAATTGACATAAAATGACCTCTTTT
FTN_0757-aptamer_2.FOR	AGAGGTCATTTTATGTCAATTTATCAAG
Aptamer.REV	GGAGAGCGTTCACCGACAAA
cas12 promoter aptamer	CTAGTAGATGAAACGCTAAAAATATATGCCCTTGCACCAATGTCTGTGGCACCAAAATATCAACATCAAGGAATTGGTTCTAAGCTTATAGAAGCAATGAT<br>TAAGGAAGCCAAAAAAAATAATATTGATGCAATATTTGTCTTAGGTCATCCAAGTTATTATCCAAAATTTGGTTTTAAACCAGCCACAGAATATCAGATAAAA<br>TGTGAATATGATGTCCCAGCGGATGTTTTTATGGTACTAGATTTGTCAGCTAAACTAGCTAGTTTAAAAGGACAAACTGTCTACTATGCCGATGAGTTTGG<br>CAAAATTTTTTAGATCTACAAAATTATAAACTAAATAAAGATTCTTATAATAACTTTATATATAATCGAAATGTAGAGAATTTTATAAGGAGTCTTTATCATGTC<br>AATTTATCAAGAATTTGTTAATAAATAGACGCGACCGAAATGGTGAAGGACGGGTCCAGTGCTTCGGCACTGTTGAGTAGAGTGTGAGCTCCGTAACTGG<br>TCGCGTCTAGTTTAAGTAAAACTCTAAGATTTGAGTTAACCAGGCATCAAATAAAACGAAAGGCTCAGTCGAAAGACTGGGCCTTTCGTTTTATCTGTTGT<br>TTGTCGGTGAACGCTCTCC
FTN_1397-aptamer_1.FOR	GCGCGCGCGCAGATGAAACGCTAAAAATATATG
FTN_1397-aptamer_1.REV	ATAAATTGACATGATAAAGACTCCTTA
FTN_1397-aptamer_2.REV	GGAGTCTTTATCATGTCAATTTATCAAG
Aptamer.REV	GGAGAGCGTTCACCGACAAA

Supplementary References

1. Baba, T., Ara, T., Hasegawa, M., Takai, Y., Okumura, Y., Baba, M., Datsenko, K. A., Tomita, M., Wanner, B. L. & Mori, H. Construction of *Escherichia coli* K-12 in-frame, single-gene knockout mutants: the Keio collection. *Mol. Syst. Biol.* **2**, 2006.0008 (2006).

2. Hossain, A. A., Pigli, Y. Z., Baca, C. F., Heissel, S., Thomas, A., Libis, V. K., Burian, J., Chappie, J. S., Brady, S. F., Rice, P. A. & Marraffini, L. A. DNA glycosylases provide antiviral defence in prokaryotes. *Nature* **629**, 410–416 (2024).

3. Harms, A., Segers, F. H. I. D., Quebatte, M., Mistl, C., Manfredi, P., Körner, J., Chomel, B. B., Kosoy, M., Maruyama, S., Engel, P. & Dehio, C. Evolutionary Dynamics of Pathoadaptation Revealed by Three Independent Acquisitions of the VirB/D4 Type IV Secretion System in *Bartonella*. *Genome Biol. Evol.* **9**, 761–776 (2017).

4. Brodmann, M., Heilig, R., Broz, P. & Basler, M. Mobilizable Plasmids for Tunable Gene Expression in *Francisella novicida*. *Front. Cell. Infect. Microbiol.* **8**, (2018).

5. Twist, K.-A. F., Campbell, E. A., Deighan, P., Nechaev, S., Jain, V., Geiduschek, E. P., Hochschild, A. & Darst, S. A. Crystal structure of the bacteriophage T4 late-transcription coactivator gp33 with the β-subunit flap domain of *Escherichia coli* RNA polymerase. *Proc. Natl. Acad. Sci. U. S. A.* **108**, 19961–19966 (2011).

6. Chen, J., Gopalkrishnan, S., Chiu, C., Chen, A. Y., Campbell, E. A., Gourse, R. L., Ross, W. & Darst, S. A. *E. coli* TraR allosterically regulates transcription initiation by altering RNA polymerase conformation. *eLife* **8**, e49375 (2019).

7. Chen, J., Wassarman, K. M., Feng, S., Leon, K., Feklistov, A., Winkelman, J. T., Li, Z., Walz, T., Campbell, E. A. & Darst, S. A. 6S RNA Mimics B-Form DNA to Regulate *Escherichia coli* RNA Polymerase. *Mol. Cell* **68**, 388-397.e6 (2017).
