# Supplementary material for: MtvS1 and MtvS2 Interact with RNA polymerase to regulate the Francisella type V-A CRISPR-Cas system
Source: Nat Commun. 2026 Sep 1;17:10400. doi: 10.1038/s41467-026-77062-6 (PMC13627669; doi:10.1038/s41467-026-77062-6)
Supplement: Supplementary file 4 [file 41467_2026_77062_MOESM2_ESM.pdf]

Description of Additional Supplementary Files:

Supplementary Data 1: contains Proteomics, RNA-seq and bioinformatics analyses belonging to main and supplementary figures.

Supplementary Movie 1: Cas12-mCherry2 is expressed at high cell density. Fluorescence microscopy of Cas12-mCherry2 in the parental strain in a microfluidic plate. BHI was flown through for 8 h. Three representative examples are shown (n = 3 biological replicates). Pictures were taken every 5 min. Same contrast settings as in Fig. 4a-b and Supplementary Movies 2-4. Merge of phase contrast and RFP channel is shown. Fields of view are 13 x 13 µm. Scalebars represent 1 µm. Movies play at a frame rate of 7 frames per second.

Supplementary Movie 2: Cas12-mCherry2 is not expressed at low cell density. Fluorescence microscopy of Cas12-mCherry2 in the parental strain in a microfluidic plate. BHI was flown through for 8 h. Three representative examples are shown (n = 3 biological replicates). Pictures were taken every 5 min. Same contrast settings as in Fig. 4a-b and Supplementary Movies 1, 3-4. Merge of phase contrast and RFP channel is shown. Fields of view are 13 x 13 µm. Scalebars represent 1 µm. Movies play at a frame rate of 7 frames per second.

Supplementary Movie 3: Cas12-mCherry2 is not expressed at high cell density in *mtvS1 nonsense* mutant. Fluorescence microscopy of Cas12-mCherry2 in the *mtvS1 nonsense* mutant in a microfluidic plate. BHI was flown through for 8 h. Three representative examples are shown (n = 3 biological replicates). Pictures were taken every 5 min. Same contrast settings as in Fig. 4a-b and Supplementary Movies 1-2, 4. Merge of phase contrast and RFP channel is shown. Fields of view are 13 x 13 µm. Scalebars represent 1 µm. Movies play at a frame rate of 7 frames per second.

Supplementary Movie 4: Cas12-mCherry2 is not expressed at low cell density in *mtvS1 nonsense* mutant. Fluorescence microscopy of Cas12-mCherry2 in the *mtvS1 nonsense* mutant in a microfluidic plate. BHI was flown through for 8 h. Three representative examples are shown (n = 3 biological replicates). Pictures were taken every 5 min. Same contrast settings as in Fig. 4a-b and Supplementary Movies 1-3. Merge of phase contrast and RFP channel is shown. Fields of view are 13 x 13 µm. Scalebars represent 1 µm. Movies play at a frame rate of 7 frames per second.

Supplementary Movie 5: *F. novicida* U112 reacts to spent overnight medium with growth arrest, morphology change and Cas12-mCherry2 expression. Fluorescence microscopy of Cas12-mCherry2 in the parental strain in a microfluidic plate. BHI was flown through for 1 h before switching to spent overnight medium of parental strain for 4h. Afterwards, fresh BHI was flown through for 3 h. Three representative examples are shown (n = 3 biological replicates). Pictures were taken every 5 min. Same contrast settings as in Figure 4cg, Supplementary Fig. 4d

and Supplementary Movies 7-8. Merge of phase contrast and RFP channel is shown. Fields of view are 13 x 13 µm. Scalebars represent 1 µm. Movies play at a frame rate of 7 frames per second.

Supplementary Movie 6: *F. novicida* U112 reacts to spent overnight medium with growth arrest, morphology change and mCherry2 expression from *cas12* promoter. Fluorescence microscopy of mCherry2 in a strain carrying pFNMB *prom-cas12 mCherry2* in a microfluidic plate. BHI was flown through for 1 h before switching to spent overnight medium of parental strain for 4h. Afterwards, fresh BHI was flown through for 3 h. Three representative examples are shown (n = 2 biological replicates). Pictures were taken every 5 min. Same contrast settings as in Supplementary Fig. 4a-c. Merge of phase contrast and RFP channel is shown. Fields of view are 13 x 13 µm. Scalebars represent 1 µm. Movies play at a frame rate of 7 frames per second.

Supplementary Movie 7: Cas12 is not expressed in *mtsvS1 nonsense* mutant in spent overnight medium. Fluorescence microscopy of Cas12-mCherry2 in the *mtvS1 nonsense* mutant in a microfluidic plate. BHI was flown through for 1 h before switching to spent overnight medium of parental strain for 4h. Afterwards, fresh BHI was flown through for 3 h. Three representative examples are shown (n = 2 biological replicates). Pictures were taken every 5 min. Same contrast settings as in Fig. 4c-g, Supplementary Fig. 4d and Supplementary Movies 5 and 8. Merge of phase contrast and RFP channel is shown. Fields of view are 13 x 13 µm. Scalebars represent 1 µm. Movies play at a frame rate of 7 frames per second.

Supplementary Movie 8: *F. novicida* U112 does not react to heat-treated spent overnight medium with morphology change and Cas12 expression. Fluorescence microscopy of Cas12-mCherry2 in the parental strain in a microfluidic plate. BHI was flown through for 1 h before switching to heat-treated spent overnight medium of parental strain for 4h. Afterwards, fresh BHI was flown through for 3 h. Three representative examples are shown (n = 2 biological replicates). Pictures were taken every 5 min. Same contrast settings as in Fig. 4c-g, Supplementary Fig. 4d and Supplementary Movies 5 and 8. Merge of phase contrast and RFP channel is shown. Fields of view are 13 x 13 µm. Scalebars represent 1 µm. Movies play at a frame rate of 7 frames per second.
